# Metabolic aspects of genetic ion channel epilepsies

**DOI:** 10.1111/jnc.15938

**Published:** 2023-08-18

**Authors:** Elliott S. Neal, Weizhi Xu, Karin Borges

**Affiliations:** ^1^ School of Biomedical Sciences The University of Queensland St Lucia Queensland Australia

**Keywords:** brain energy metabolism, Dravet syndrome, KCNA1, Kv1.1, Nav1.1, SCN1A

## Abstract

Nowadays, particularly in countries with high incomes, individual mutations in people affected by genetic epilepsies are identified, and genetic therapies are being developed. In addition, drugs are being screened to directly target specific mutations, and personalised medicine is possible. However, people with epilepsy do not yet benefit from these advances, and many types of epilepsies are medication‐resistant, including Dravet syndrome. Thus, in the meantime, alternative and effective treatment options are needed. There is increasing evidence that metabolic deficits contribute to epileptic seizures and that such metabolic impairments may be amenable to treatment, with metabolic treatment options like the ketogenic diet being employed with some success. However, the brain metabolic alterations that occur in ion channel epilepsies are not well‐understood, nor how these may differ from epilepsies that are of acquired and unknown origins. Here, we provide an overview of studies investigating metabolic alterations in epilepsies caused by mutations in the *SCN1A* and *KCNA1* genes, which are currently the most studied ion channel epilepsies in animal models. The metabolic changes found in these models are likely to contribute to seizures. A metabolic basis of these ion channel epilepsies is supported by human and/or animal studies that show beneficial effects of the ketogenic diet, which may be mediated by the provision of auxiliary brain fuel in the form of ketone bodies. Other potentially more preferred dietary therapies including medium‐chain triglycerides and triheptanoin have also been tested in a limited number of studies, but their efficacies remain to be clearly established. The extent to which brain metabolism is affected in people with Dravet syndrome, *KCNA1* epilepsy and the models thereof still requires clarification. This requires more experiments that yield functional insight into metabolism.

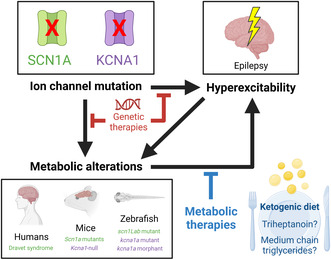

## Graphical abstract

There is increasing awareness of contributions of impaired brain metabolism to epilepsies. We review existing studies regarding metabolism in epilepsies caused by mutations in sodium (SCN1A) and potassium (KCNA1) channels. This is important as gene therapy is not yet available. While there are still little data about impairments of brain metabolism, the studies revealed ketogenic diets to be mostly effective, particularly for Dravet syndrome with identified SCN1A mutations as well as mouse and zebrafish models with SCN1A and KCNA1 mutations. We discuss how ketogenic diet and potentially other metabolic therapies may help to prevent seizures in these ion channel epilepsies.

**Abbreviations**
2DG: 2‐deoxyglucose
4‐AP: 4‐aminopyridine
6PG: 6‐phosphogluconate
6PGDL: 6‐phosphogluconolactone
ACAT1: acetyl‐CoA acetyltransferase 1
ADP: adenosine diphosphate
αKG: alpha‐ketoglutarate
AMPA: α‐amino‐3‐hydroxy‐5‐methyl‐4‐isoxazolepropionic acid
Asp: aspartate
ATP: adenosine triphosphate
βHB: beta‐hydroxybutyrate
CMR_glc_
: cerebral metabolic rate for glucose
DHAP: dihydroxyacetone phosphate
dpf: days post‐fertilisation
EA1: episodic ataxia type 1
ECAR: extracellular acidification rate
EEG: electroencephalogram
ECF: extracellular fluid
ETC: electron transport chain
F16BP: fructose‐1,6‐bisphosphate
F6P: fructose‐6‐phosphate
FAD: flavin adenine dinucleotide
FADH_2_
: reduced flavin adenine dinucleotide
FDG: ^18^F‐fluorodeoxyglucose
FDG6P: ^18^F‐fluorodeoxyglucose‐6‐phosphate
G1P: glucose‐1‐phosphate
G6P: glucose‐6‐phosphate
*g6pca.1*
: glucose‐6‐phosphate catalytic subunit 1A
G6PDH: glucose‐6‐phosphate dehydrogenase
GA3P: glyceraldehyde 3‐phosphate
GABA: gamma‐aminobutyric acid
*gad1b*
: glutamate decarboxylase 1b
GEFS+: epilepsy with febrile seizures plus
Gln: glutamine
GlnS: glutamine synthetase
Glu: glutamate
GLUT: glucose transporter
GP: glycogen phosphorylase
GPI: glucose‐6‐phosphate isomerase
GYS: glycogen synthase
HK1: hexokinase 1
HMGCS2: 3‐hydroxy‐3‐methylglutaryl‐CoA synthase‐2
IDH3A: isocitrate dehydrogenase [NAD] subunit alpha
KCNA1: potassium voltage‐gated channel subfamily A member 1
KD: ketogenic diet
LDH: lactate dehydrogenase
MCFA: medium‐chain fatty acid
MCT1: monocarboxylate transporter 1
MDH2: malate dehydrogenase 2
NAD+: nicotinamide adenine dinucleotide
NADH: reduced nicotinamide adenine dinucleotide
NADP+: nicotinamide adenine dinucleotide phosphate
NADPH: reduced nicotinamide adenine dinucleotide phosphate
OAA: oxaloacetate
OCR: oxygen consumption rate
OGDH: 2‐oxoglutarate dehydrogenase
Oxphos: oxidative phosphorylation
PC: pyruvate carboxylase
PCK (*pck*) 1/2: Phosphoenolpyruvate carboxykinase 1/2
PDH: pyruvate dehydrogenase
PDHA1: pyruvate dehydrogenase E1 subunit alpha 1
PDK (*pdk*): pyruvate dehydrogenase kinase
PEP: phosphoenolpyruvate
PET: positron emission tomography
PGM: phosphoglucomutase
*phkg1a*
: phosphorylase gamma kinase 1a
Pi: inorganic phosphate
PPP: pentose phosphate pathway
PTZ: Pentylenetetrazol
RL5P: ribulose 5‐phosphate
SCN1A: sodium voltage‐gated channel alpha subunit 1
SCOT: succinyl‐CoA:3‐ketoacid CoA transferase
SDHA/B: succinate dehydrogenase complex flavoprotein subunit A/B
SMEI: severe myoclonic epilepsy in infancy
SUCLA2: succinate‐CoA ligase subunit beta
SUCLG1: succinate‐CoA ligase subunit alpha
SUDEP: sudden unexpected death in epilepsy
SUV: standardised uptake value
TCA: tricarboxylic acid
UCP: mitochondrial uncoupling protein
*vglut2a*
: vesicular glutamate transporter 2

## 1 INTRODUCTION

Much of the research in the epilepsy field has focused on aberrant neuronal activity, and this has led to the development of many anti‐seizure drugs which mostly reduce neuronal (hyper‐) excitability (Rogawski et al., 2016; Sills & Rogawski, 2020; White et al., 2007). However, there is now increasing evidence to show that brain metabolic dysfunction may contribute to epilepsy (Kudin et al., 2009; Patel, 2018; Reid et al., 2014; Rho & Boison, 2022). One argument for the notion that metabolism plays a role in seizure generation is that the ketogenic diet is perhaps the most effective way to control seizures in some treatment‐resistant epilepsies, including Dravet syndrome (Knupp & Wirrell, 2018). On the other hand, Dravet syndrome is caused by in most cases known mutations in the voltage‐gated sodium channel gene *SCN1A*. Thus, questions arise as to why and how metabolism is altered in genetic ion channel epilepsies, and whether alterations in metabolism contribute to seizure generation in these disorders. Studies assessing metabolic therapies in these disorders are limited. This could be explained by the fact that current research is now increasingly directed at personalised medicine targeting specific ion channel gain or loss of functions, or even specific mutations in these ion channels, for which gene therapies are being developed. Increasing *SCN1A* expression with antisense oligonucleotides or with AAV‐9‐based gene therapy has been successful in mouse models of Dravet syndrome (Han et al., 2020; Lim et al., 2020; Tanenhaus et al., 2022), and trials in humans are underway (Sullivan & Wirrell, 2023). However, people affected by genetic epilepsies require urgent treatment, and current medications are often ineffective, such as in about 30–70% of people affected by Dravet syndrome (see Table 2 in Wirrell and Nabbout (2019)). Ketogenic diet is often used specifically to treat Dravet syndrome and appears to be the most effective treatment for this ion channel disorder (Knupp & Wirrell, 2018; Wang et al., 2020). However, many affected people find this strict high‐fat diet difficult to follow, and easier metabolic treatment options are preferred. Thus, research into metabolic alterations in Dravet syndrome is important to enable development of new approaches for treatment. Interestingly, the effects of the ketogenic diet have also been researched in animals with mutations in the potassium channel gene *KCNA1*, but to our knowledge, people with *KCNA1*‐associated epilepsy (hereon referred to as *KCNA1* epilepsy) are not treated with this dietary regimen.

This review summarises metabolic changes that have been found in people with epilepsy and in rodent seizure and epilepsy models, what is currently known about metabolism in specific epilepsies associated with mutations in *SCN1A* and *KCNA1*, and alterations found after ketogenic diet treatment. While there are many genetic epilepsy syndromes linked to mutations in other ion channel genes (reviewed in Nicita et al. (2012), Lerche et al. (2013) and Zimmern et al. (2022)), only case reports were found regarding metabolism, and only a sufficient number of studies warranting discussion were available for epilepsies linked to mutations in *SCN1A* and *KCNA1* at this time.

## 2 ENERGY METABOLISM DEFICITS IN THE EPILEPTIC BRAIN

### 2.1 Metabolism of the main fuels used in brain

**FIGURE 1:**
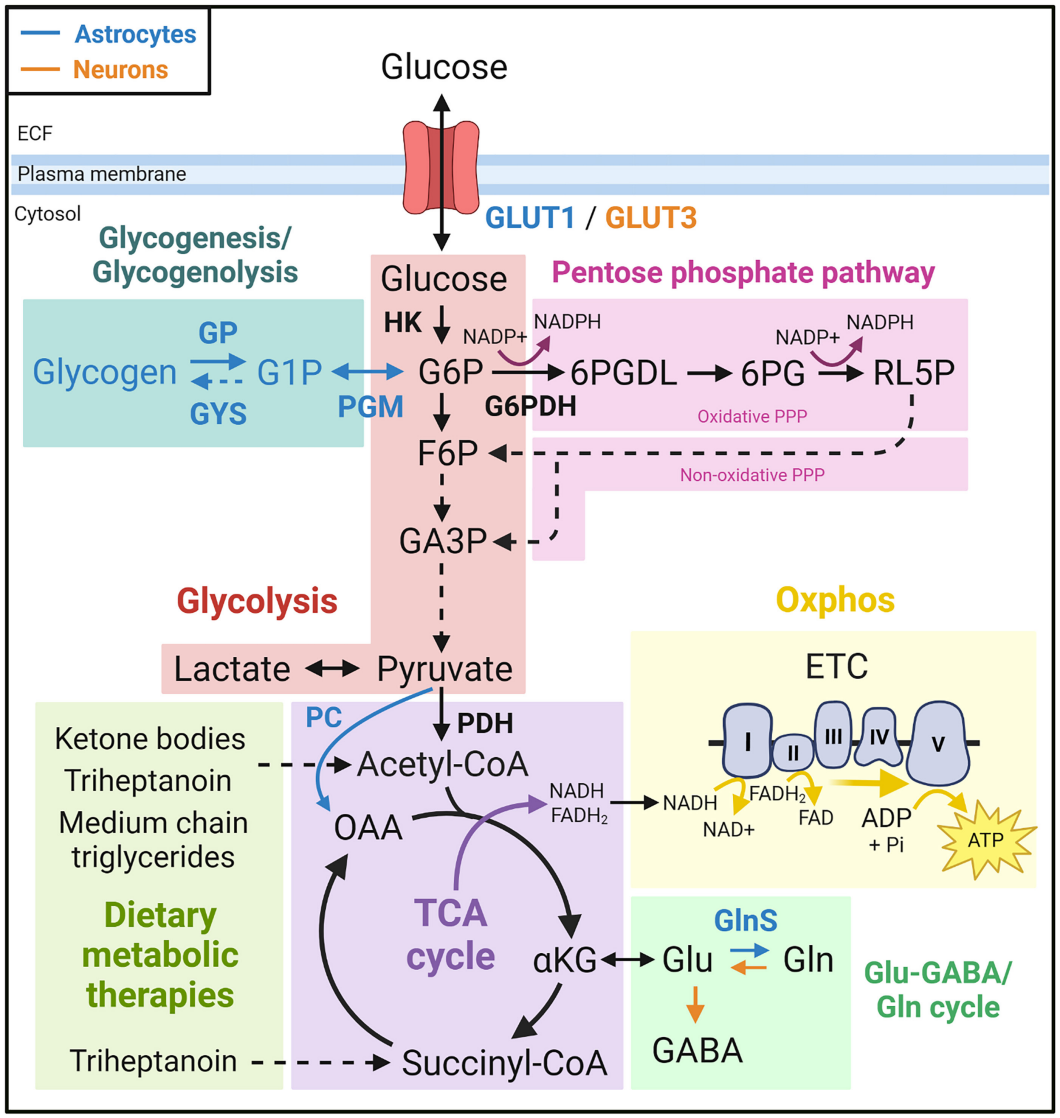
Simplified overview of energy metabolism in the brain. A summary of glucose metabolism via glycolysis, pentose phosphate pathway and the TCA cycle is given. Glucose carbons can also be stored as glycogen or metabolised into amino acids. Dietary metabolic therapies (ketogenic diets, triheptanoin and medium‐chain triglycerides) provide auxiliary brain fuels that can be used in brain to synthesise acetyl‐CoA and succinyl‐CoA, independent from pyruvate dehydrogenase (PDH). Blue arrows or texts indicate that the pathway is largely astrocytic, while orange arrows or texts indicate that the process is predominantly neuronal. 6PG, 6‐phosphogluconate; 6PGDL, 6‐phosphogluconolactone; ADP, adenosine diphosphate; ATP, adenosine triphosphate; ECF, extracellular fluid; ETC, electron transport chain; F6P, fructose‐6‐phosphate; FAD, flavin adenine dinucleotide; FADH_2_, reduced flavin adenine dinucleotide; G1P, glucose‐1‐phosphate; G6P, glucose‐6‐phosphate; G6PDH, glucose‐6‐phosphate dehydrogenase; GA3P, glyceraldehyde 3‐phosphate; GABA, gamma‐aminobutyric acid; Gln, glutamine; GlnS, glutamine synthetase; Glu, glutamate; GLUT1, glucose transporter 1; GLUT3, glucose transporter 3; GP, glycogen phosphorylase; GYS, glycogen synthase; HK, hexokinase; NAD+, nicotinamide adenine dinucleotide; NADH, reduced nicotinamide adenine dinucleotide; NADP+, nicotinamide adenine dinucleotide phosphate; NADPH, reduced nicotinamide adenine dinucleotide phosphate; OAA, oxaloacetate; Oxphos, oxidative phosphorylation; PC, pyruvate carboxylase; PGM, phosphoglucomutase; Pi, inorganic phosphate; PPP, pentose phosphate pathway; RL5P, ribulose 5‐phosphate; TCA, tricarboxylic acid; αKG, alpha‐ketoglutarate.

A short, simplified overview of energy metabolism in the brain is provided (Figure 1). The main brain fuel is glucose, which is broken down via glycolysis to pyruvate. While most of the metabolised glucose enters glycolysis, some may go into the pentose phosphate pathway to produce cytosolic NADPH and riboses. Alternatively, glucose can be metabolised to glycogen, which occurs mostly in astrocytes. Glycogen is used as a fuel when quick energy is needed—for example, during brain stimulation, in response to stress, and also during seizures. Interestingly, glycogen is then metabolised mostly to lactate. In the absence of oxygen or when the brain is highly active, pyruvate produced from glucose is metabolised to lactate. Otherwise, pyruvate can enter mitochondria via pyruvate transporters where it is then metabolised via the tricarboxylic acid (TCA) cycle to yield some ATP, as well as the reducing equivalents NADH and FADH_2_, which are used in oxidative phosphorylation to produce much higher amounts of ATP. Pyruvate can also be converted into oxaloacetate via pyruvate carboxylase, which occurs mostly in astrocytes. Other fuels that can be used by brain are the C4 ketone bodies, beta‐hydroxybutyrate (βHB) and acetoacetate, as well as medium‐chain fatty acids (MCFAs). These do not rely on metabolism by pyruvate dehydrogenase (PDH), an enzyme that is susceptible to inhibition by oxidative stress (Martin et al., 2005). As PDH activity was shown to be reduced in mice in the chronic stage of the pilocarpine epilepsy model (Durie et al., 2018; McDonald et al., 2017) and also after acute flurothyl‐induced seizures (McDonald & Borges, 2017), and oxidative stress is associated with frequent seizures (reviewed in Dienel et al. (2023)), ketone bodies and MCFAs are thus ideal high energy fuels for epileptogenic brain areas, which experience fuel shortages that may contribute to seizures (see Section 2.3). These fuels can be provided via dietary metabolic therapies, such as the ketogenic diet, medium‐chain triglycerides and triheptanoin.

### 2.2 Provision of auxiliary brain fuels by dietary metabolic therapies

Ketogenic diets raise blood βHB and acetoacetate levels, which can be converted via ketolysis in brain into acetyl‐CoA and used in the TCA cycle. Commercially available medium‐chain triglycerides usually contain the MCFAs octanoic (C8) and decanoic (C10) acid (see Section 5.4). Once consumed, medium‐chain triglycerides are broken down in the gastrointestinal tract into their constituent MCFAs, which can be converted within tissues to acetyl‐CoA via beta‐oxidation. MCFAs provided by medium‐chain triglycerides can also be used as a substrate for ketogenesis, and thus used for synthesis of C4 ketone bodies in the liver, particularly when blood glucose levels are low. After release into the blood, ketone bodies provide a source of acetyl‐CoA in extra‐hepatic tissues. Triheptanoin, the uneven medium‐chain triglyceride of heptanoic acid (C7), is broken down in the gastrointestinal tract into the uneven MCFA heptanoate (C7), which uniquely can provide both acetyl‐CoA and succinyl‐CoA (for anaplerosis via propionyl‐CoA, produced from beta‐oxidation of heptanoic acid). Heptanoate can also be used in the liver to synthesise the C5 ketone bodies β‐ketopentanoate and β‐hydroxypentanoate, which are additional sources of acetyl‐ and succinyl‐CoA (see Figure 1).

### 2.3 Impaired energy metabolism and seizure generation

Synaptic signalling, action potentials and maintenance of resting potentials including potassium uptake are the most energy‐expensive processes in the brain (Barros, 2022; Engl & Attwell, 2015). Glucose and astrocytic glycogen are the main substrates that fuel brain activity, and sufficient energy is needed to prevent seizure generation. During stimulation and/or physiological increases in brain activity, cerebral blood flow and glucose utilisation are enhanced and glycogen breaks down to provide quick additional ATP (Dienel, 2019a). Epileptic phenotypes are common in people and in animals with deficits in energy metabolism pathways, such as deficiencies in subunits of the pyruvate dehydrogenase complex (PDH; Jakkamsetti et al., 2019; Prasad et al., 2011) or in glucose transporter 1 (GLUT1) (Striano et al., 2012), the main glucose transporter at the blood–brain barrier. Furthermore, chronic partial inhibition of brain glycolysis in rats in vivo, via daily intracerebroventricular (i.c.v.) injections of 2‐deoxyglucose (2DG), induced epileptiform activity, increased the severity of pentylenetetrazol (PTZ)‐induced seizures and caused neuronal cell loss and mossy fibre sprouting in the hippocampus, which are structural changes that are consistent with epileptogenesis (Samokhina et al., 2017, 2020). Energy shortages may occur during periods when brain energy needs are high, such as brain stimulation (Rothman et al., 2022), which would explain why seizures more commonly occur with certain sensory stimulations or during stress (McKee & Privitera, 2017). Low energy availability is expected to impair the most energy‐expensive processes in the brain, such as the fuelling of the Na^+^/K^+^‐ATPase, which is needed for maintenance of resting potentials and for potassium buffering (Barros, 2022; Engl & Attwell, 2015). If Na^+^/K^+^‐ATPase activity is insufficient, this will increase levels of extracellular potassium and/or cause other potential local ion imbalances in small neuronal processes, which can lead to neuronal membrane depolarisation and seizure generation (Bergmann et al., 1970).

Further evidence for a role of impaired glucose metabolism in seizure generation comes from the anticonvulsant effects of auxiliary brain fuels, which provide additional energy to the brain, independent of glycolysis. For people with epilepsy, this includes ketone bodies, mostly βHB and acetoacetate, produced by the liver during fasting and/or while on a ketogenic diet (Cunnane et al., 2020; Masino, 2022; Masino & Rho, 2019), and potentially medium‐chain fatty acids derived from medium‐chain triglycerides or triheptanoin (Borges, 2022; Han, Conboy‐Schmidt, et al., 2021; Schoeler et al., 2021; Striano et al., 2022). In animal models, anticonvulsant effects of pyruvate are also found, when administered alone (Popova et al., 2017) or as a cocktail with ascorbic acid and alpha‐tocopherol (Simeone et al., 2014). Anticonvulsant effects of TCA cycle intermediates that are anaplerotic were also found, including alpha‐ketoglutarate, oxaloacetate, succinate and succinyl‐CoA (as provided by triheptanoin) (reviewed in Kovac et al. (2013), McDonald et al. (2018) and Borges (2022)). On the other hand, anticonvulsant effects of 2DG administration (Garriga‐Canut et al., 2006; Gasior et al., 2010; Stafstrom et al., 2009) and inhibition of lactate dehydrogenase (LDH) (Sada et al., 2015) have been reported, in addition to proconvulsant effects of pyruvate (Gonzalez et al., 2005) and succinate (Roehrs et al., 2004), but we note that alternative explanations for some of these effects have been proposed (Kovac et al., 2013; Samokhina et al., 2017, 2020). For instance, in the studies that found anticonvulsant actions of 2DG administration in vivo, 2DG was delivered via intraperitoneal (i.p.) injection. This was found to be protective against seizures at doses of ~80–500 mg/kg when given shortly prior to seizure tests (15 min ‐ 4 h prior), including in rat kindling models, mouse 6 Hz and acute pilocarpine seizure models, and Fring's audiogenic seizure‐susceptible mice (Garriga‐Canut et al., 2006; Gasior et al., 2010; Lian et al., 2007; Stafstrom et al., 2009). However, Samokhina et al. (2017) calculated that even at the highest doses used in those studies (500 mg/kg 2DG, i.p.), this would only have achieved ~2% inhibition of brain glycolysis. This is sevenfold lower than the 14% inhibition calculated for their 2.5 μL i.c.v. injections of 20 mM 2DG used in their own study (Samokhina et al., 2017). They further reasoned that 2DG injections raise blood glucose levels and cerebral blood flow in rats (Breier et al., 1993; Combs et al., 1986; Horinaka et al., 1997) and humans (Elman et al., 1999; Landau et al., 1958), and these effects most likely explain the anticonvulsant actions of acute i.p. 2DG seen in some tests (Samokhina et al., 2017, 2020). We also note that no or proconvulsant effects of 2DG were found in other acute seizure tests, when seizures were induced by maximal electroshock, PTZ and kainic acid (Gasior et al., 2010; Stafstrom et al., 2009). In addition, while the anticonvulsant mechanisms of the ketogenic diet appear to be multi‐pronged (for example, see Masino and Rho (2012), Rogawski et al. (2016), Youngson et al. (2017), Masino and Rho (2019), Sourbron et al. (2021) and Masino (2022)), one proposed mechanism of action of this diet is, similar to above, inhibition of brain glycolysis (Rho et al., 2019; Shao et al., 2018; Stafstrom et al., 2009) and/or overall reduced brain glucose utilisation (Lian et al. (2007); reviewed in Zilberter and Zilberter (2020) and Cunnane et al. (2020)). However, an alternative interpretation of a role of reduced glucose metabolism is that the ketogenic diet provides additional fuel in the form of ketone bodies that reduces the need for brain oxidative glucose utilisation (Zhang et al., 2013, 2015). Consistent with this, rats fed a ketogenic diet for ~3–4 weeks showed no difference in ATP concentrations measured in microwave‐fixed hippocampal tissue compared to rats fed a control diet (Bough et al., 2006). Interestingly in a meta‐analysis of 11 human or rat studies by Zhang et al. (2013), it was found that with every 1 mM increase in blood ketone body levels, the brain cerebral metabolic rate for glucose (CMR_glc_) decreased by 9%. Use of ketones as fuel could spare glucose for other functions, where glucose is required and cannot be replaced by alternative fuels, such as for the generation of NADPH via the pentose phosphate pathway, which is needed to keep glutathione in its reduced antioxidant state (Zilberter et al., 2022; Zilberter & Zilberter, 2020). This hypothesis is also supported by the anticonvulsant effects of fructose‐1,6‐bisphosphate (F16BP) found in many seizure and epilepsy models (Janicot et al., 2020; McDonald et al., 2021; Stringer & Xu, 2008). In the chronic pilocarpine model, F16BP increased oxidative glucose metabolism (McDonald et al., 2021), which might reduce the need to produce ATP via glycolysis and thus spare endogenous glucose for metabolism via the pentose phosphate pathway.

### 2.4 Low interictal ^18^F‐FDG‐PET signals in epileptogenic brain areas

^18^F‐fluorodeoxyglucose (FDG) positron emission tomography (PET) is a tool that is used clinically and in animal models to assess in vivo rates of glucose utilisation. In people and in animal models of chronic epilepsy, epileptogenic brain areas, and sometimes surrounding tissue involved in seizure propagation, show low FDG‐PET signals in the interictal period (in between seizures), which are interpreted as glucose hypometabolism (summarised by Dienel et al. (2023)). In contrast, during seizures, energy needs are high and FDG‐PET signals are increased in areas showing high electrical activity (Schur et al., 2018). After a bolus injection, the radioactive glucose analogue FDG is taken up via glucose transporters at the blood–brain barrier and into brain cells. FDG is quickly phosphorylated by hexokinase to produce FDG‐6‐phosphate, which gets trapped, as it cannot undergo further metabolism via glycolysis. This is due to the fact that in FDG, the 2‐hydroxyl group needed for further phosphorylation is replaced by ^18^F. Thus, FDG‐PET signal intensity corresponds to the amount of radiation produced from largely FDG‐6‐phosphate and reflects the rate of glucose transport plus hexokinase activity. In cases where morphological alterations such as neuronal loss and gliosis cannot explain the low interictal FDG‐PET signals in epilepsy, the mechanisms for this phenomenon have yet to be identified. It is not explained by decreased transport of glucose via the blood–brain barrier, as reports have shown normal brain glucose levels in people with epilepsy (reviewed in Dienel et al. (2023)), and in several cases, increased vascularisation in epileptogenic areas in people with temporal lobe epilepsy and in animal models (reviewed in van Lanen et al. (2021)). We have recently proposed increased glycogen utilisation as a possible explanation for the low FDG‐PET signals in epileptogenic brain areas, which would reduce the use of glucose as fuel (Dienel et al., 2023).

### 2.5 Other metabolic alterations in epileptogenic brain areas

Detailed reviews of alterations to cytosolic and mitochondrial glucose metabolism as well as other alterations in the electron transport chain in epileptogenic brain areas in humans and animal models can be found in our previous works (Han, McDonald, et al., 2021; McDonald et al., 2018). Considerably more is known about deficits in oxidative glucose metabolism in brain in animal models of chronic epilepsy as compared to glycolysis (McDonald et al., 2018), potentially due to greater interest in mitochondria, which produce the majority of ATP in the TCA cycle and electron transport chain as compared to glycolysis. In a recent study from our group, the total amounts of all metabolites were similar, while enrichment of ^13^C from [U‐^13^C]glucose (i.p.) into glycolytic intermediates in the hippocampal formation was reduced in the chronic stage of the mouse pilocarpine model of epilepsy (glucose‐6‐phosphate (G6P), fructose‐6‐phosphate (F6P), dihydroxyacetone phosphate (DHAP) and phosphoenolpyruvate (PEP), 17–22% lower; McDonald et al., 2017). In that study, ^13^C enrichment into G6P was highly correlated with ^13^C enrichment in all downstream metabolites of glycolysis, and maximal activities of all cytosolic enzymes tested were unaltered. This indicated that there were no deficits in the activity of glycolysis per se, at least after the hexokinase step. We have considered that this could be explained by decreased glucose uptake (McDonald et al., 2017). However, as the amounts of total glucose and ^13^C glucose in several brain areas and the enrichment of ^13^C into plasma glucose all remained similar in our chronic epileptic mice (Smeland et al. (2013); McDonald et al. (2020); and new, unpublished data from our lab), another potential and more likely explanation is dilution of the ^13^C label from an unlabelled carbon source, such as the unlabelled G6P produced from the breakdown of glycogen (Dienel et al., 2023; McDonald et al., 2020). However, at the time of these studies (McDonald et al., 2017, 2020), we had not assessed brain glycogen content in this model, nor had others, in any chronic epilepsy model.

Brain glycogen is stored predominantly in astrocytes and functions as a reserve of quickly accessible fuel that can be used to supplement glucose when energy needs are high (Dienel, 2019a). In the rested state, brain largely metabolises glucose by glycolysis and oxidative phosphorylation for generation of ATP. Interestingly, during periods of physiological high neuronal activity as well as seizures, glycolysis of glucose and glycogen‐derived hexose‐phosphates increases more than oxidative glucose metabolism leading to enhanced production of lactate, which is excreted from the brain to a large extent (Dienel, 2019a). It is thought that glycolysis is specifically important during brain stimulation, as it quickly provides ATP in the cytosol to fuel Na^+^/K^+^‐ATPase activity and other enzymatic processes involved in neuronal signalling, although glycolysis produces about tenfold less ATP than oxidative glucose metabolism (Hertz et al., 2007). It is specifically advantageous to use stored glycogen as compared to glucose, because glycogen is metabolised to G6P (via glucose‐1‐phosphate, G1P) without the need for ATP and hexokinase. Thus, glycogen can more quickly provide ATP and yields 50% more ATP via glycolysis as compared to metabolism of glucose (3 vs. 2 ATP) (Hertz et al., 2007), rendering it an ideal quick fuel for astrocytic uptake of potassium via the Na^+^/K^+^‐ATPase and also glutamate uptake and metabolism when brain activity is high. Indeed, brain glycogen content decreases within minutes after acute seizures induced in rodents by chemoconvulsant chemicals or by electroshock (reviewed in Dienel et al. (2023)). Interestingly, brain glycogen content is reported to be higher in chronic epilepsy based on amounts measured in surgically excised ictogenic hippocampal foci from people (Dalsgaard et al., 2007), and more recently we showed that this is also the case in the interictal chronic stage of the mouse pilocarpine model of epilepsy in the cortex and hippocampus (Seo et al., 2022). These findings indicate long‐term interictal changes to glycogen metabolism in the epileptic brain, which we believe to be adaptive, as it may support the high energetic demands of epileptogenic tissue that show high‐frequency oscillations and/or interictal spiking and may prevent seizure generation (Dienel et al., 2023; Seo et al., 2022). On the other hand, as glycogen is also used as a fuel for abnormal neural activity once seizures have been initiated, it is unknown if higher glycogen levels may also increase the duration of seizures. Another topic of interest is the role of glycogen in neurons, and we point to studies investigating seizure susceptibility in mice with brain‐, neuron‐ and astrocytic‐specific reductions in glycogen synthase (see, for example, Duran et al. (2019), Duran et al. (2020)).

## 3 METABOLIC ASPECTS OF DRAVET SYNDROME

### 3.1 Dravet syndrome and animal models thereof

Dravet syndrome, also previously known as severe myoclonic epilepsy in infancy (SMEI), is a severe childhood epilepsy that is caused in around 80–90% of people by loss of function mutations in *SCN1A* (summarised in Scheffer et al. (2009); also see Rosander and Hallböök (2015)). People affected by Dravet syndrome usually experience frequent spontaneous recurrent seizures that are commonly medication resistant, are developmentally delayed, and are at high risk of premature death (Dravet, 2011; Mantegazza & Broccoli, 2019), most often due to sudden unexpected death in epilepsy (SUDEP) (Cooper et al., 2016). The *SCN1A* gene encodes the alpha subunit of Nav1.1, a voltage‐gated sodium channel. *SCN1A* is the most commonly mutated gene in genetic sodium channel epilepsies, and the syndromes that arise from mutations in *SCN1A* and the animal models thereof are comprehensively reviewed elsewhere (Catterall et al., 2010; Mantegazza et al., 2021; Mantegazza & Broccoli, 2019). Loss of function *SCN1A* mutations are associated with a broad spectrum of phenotypes in people that vary in severity, from ‘just’ febrile seizures, or epilepsy with febrile seizures plus (GEFS+), which are both relatively milder, to Dravet syndrome, the most severe (Catterall et al., 2010; Mantegazza & Broccoli, 2019). The aetiology of GEFS+ is more complex than Dravet syndrome, as only ~10% of people with GEFS+ are found to have an *SCN1A* mutation (Marini et al., 2007; Wallace et al., 2001). Thus, mouse models lacking functional *Scn1a*, and zebrafish models lacking the orthologous gene *scn1Lab*, are usually described as models of Dravet syndrome (as we have done so here for simplicity), but findings in these models may also apply to patients with *SCN1A*‐associated febrile seizures and GEFS+. At first sight, loss of function of a sodium channel like Nav1.1 would be expected to dampen neuronal activity. However, Nav1.1 is the major sodium channel in interneurons, and a loss of function of this channel in GABAergic interneurons explains the hyperexcitability found in Dravet syndrome (Catterall et al., 2010; Mantegazza et al., 2021; Mantegazza & Broccoli, 2019). First‐line medications are valproic acid and clobazam with efficacy in about 22–48% and 28% of people with Dravet syndrome, respectively, and topiramate and levetiracetam are also used (Knupp & Wirrell, 2018). Add‐on stiripentol and more recently add‐on fenfluramine as well as cannabidiol are also found to be effective for Dravet syndrome and are now approved in several countries. The responder rate to ketogenic diet seems to be higher, at 53–78%, although many studies do not consider the number of people that cannot tolerate or adhere to this diet and ketogenic diet is often used as an add‐on treatment to standard anti‐seizure drugs (Knupp & Wirrell, 2018; Ko et al., 2018; Strzelczyk & Schubert‐Bast, 2022; Wang et al., 2020; Wirrell & Nabbout, 2019). Importantly, among many different childhood epilepsy syndromes and conditions, Dravet syndrome is considered to respond especially well to ketogenic diet (Kossoff et al., 2018). Ketogenic diet can allow the number of anti‐seizure drugs used to be reduced, as was recently reported in a retrospective study in children with different epilepsy types (Gogou et al., 2023). Opportunities to minimise the number of anti‐seizure drugs are useful, as the use of anti‐seizure drugs can be associated with undesirable side effects, including cognitive impairment, weight gain, and depression, as well as potential drug–drug interactions, and thus often reduce quality of life.

Several FDG‐PET studies in humans and the fact that ketogenic diet is effective in some people with Dravet syndrome indicate that metabolic alterations in this disorder may contribute to seizures. Here, we review the available data from humans and from detailed studies in two independently generated *Scn1a* ^WT/A1783V^ mouse models carrying a common clinically relevant mutation in *Scn1a* and in larval zebrafish deficient in the orthologous gene *scn1Lab*. The studies in mice are from two groups, both using *Scn1a* ^WT/A1783V^ mouse models, either in a pure C57BL/6J background (Ricobaraza et al., 2019) or in a 50:50 C57BL/6J and 129S6 background (Miljanovic, Hauck, et al., 2021; Miljanovic, van Dijk, et al., 2021), both with confirmed electrographic and behavioural motor seizures and increased susceptibility to hyperthermia‐induced seizures (Miljanovic, Hauck, et al., 2021; Miljanovic, van Dijk, et al., 2021; Ricobaraza et al., 2019). Seizure frequency was reported to be seven seizures per week in the Dravet mice in the mixed background (Miljanovic, van Dijk, et al., 2021). Both groups found high early mortalities of 26% and 40% in the pure vs. the mixed background, respectively, at around the time of weaning, potentially due to SUDEP. While Ricobaraza et al. (2019) assessed brain glucose utilisation via FDG‐PET in adult mice, the studies by Miljanovic et al. undertook metabolomic and proteomic analyses in their *Scn1a* ^WT/A1783V^ mice in hippocampus in adult (~22 weeks old) and young (4 weeks old) mice, respectively (Miljanovic, Hauck, et al., 2021; Miljanovic, van Dijk, et al., 2021). Here, we review the reported expression changes in glucose transporters and enzymes as well as the levels of glucose and other metabolites in brain. Two metabolic studies are also published from one group using *scn1Lab* zebrafish larvae, another model of Dravet syndrome, which showed about 12–60 spontaneous ictal‐like events per hour (Banerji et al., 2021; Kumar et al., 2016).

### 3.2 ^18^F‐FDG‐PET studies in humans and mice

Widespread cortical low FDG‐PET signals have been identified in people with Dravet syndrome. Ferrie et al. (1996) found that in a small cohort of children with Dravet syndrome (*n* = 8, median age: 9 years old, range: 3–12 years old, combining those classified by the authors as having typical and atypical presentation), 50% exhibited some degree of interictal hypometabolism. In this study, children were diagnosed with Dravet syndrome based on a clinical examination that included sleep and video‐EEG recordings, according to standard guidelines at the time of publication. Similar findings have been reported in more recent FDG‐PET studies of people with genetically confirmed Dravet syndrome (Haginoya et al., 2018; Kumar et al., 2018). Haginoya et al. (2018) compared FDG‐PET scans in people with Dravet syndrome with confirmed *SCN1A* variants (*n* = 8, median age: 4.5 years old, range: 2–29 years old) to a diseased control group that comprised people with epilepsy with normal FDG‐PET scans (*n* = 8, median age: 9 years old, range: 4–16 years old). The Dravet group was separated into two equal subgroups based on age (≤3 years old, *n* = 4 vs. ≥6 years old, *n* = 4). Compared to the diseased controls, FDG‐PET signals were reduced in the ≥6 years old group only, in all four cortical regions, suggesting that the appearance of low FDG‐PET signals in people with Dravet syndrome may depend on age. Note that in the study by Haginoya et al. (2018), EEG was not conducted at the time of FDG‐PET acquisition. This age‐related phenomenon is corroborated by Kumar et al. (2018), who showed that FDG‐PET signals in the interictal period (confirmed by simultaneous EEG during the FDG uptake period) were normal in children with Dravet syndrome with confirmed *SCN1A* variants aged 6 months to 1 year (*n* = 3) compared to age‐matched epileptic controls (*n* = 3), but when the children with Dravet syndrome were followed up at age 3–5 years, cortical FDG‐PET signals were found to be lower compared to a separate group of age‐matched epileptic controls (*n* = 3). 18–30% reductions in FDG‐PET signals were seen in the cortical regions, with the largest decreases evident in the frontal, parietal and temporal cortex – no differences were seen in the thalamus, basal ganglia or cerebellum. Although it is unknown why the reduced FDG‐PET signals appear only in the later stages of this disorder, this has been proposed to be linked to delayed development and progression of the disease (Haginoya et al., 2018; Kumar et al., 2018). Together, findings from human studies indicate that interictal cortical FDG‐PET signals are low in Dravet syndrome, which is thought to reflect reduced cerebral glucose uptake and/or metabolism. As highlighted in our recent hypothesis paper, more studies are needed to explain the mechanism behind the conundrum that interictal FDG‐PET signals are low in certain brain areas in people with epilepsy, as interictal epileptiform activity is expected to require a lot of energy (Dienel et al., 2023).

Only one study has performed FDG‐PET in Dravet mice, which in contrast to the human studies above, reported increased brain FDG‐PET signals compared to age‐matched littermate controls (Ricobaraza et al., 2019). Higher signals (increased SUV ratios, see below) were found in both young (1–2 months) and middle‐aged (5–8 months) *Scn1a* ^WT/A1783V^ mice. Ex vivo quantification of radioisotope levels in the 5–8‐month‐old mice revealed increased tracer incorporation in all regions assessed, including the cortex, hippocampus, cerebellum and basal ganglia relative to liver. The authors attributed their finding of glucose hypermetabolism to unbalanced excitatory activity in the *Scn1a* ^WT/A1783V^ mice, which was evident in EEG recordings within the prefrontal cortex, the dentate gyrus and hippocampal CA1 area. The high amplitude electrical activity was classified as high‐frequency interictal epileptiform discharges, as there were no behavioural motor seizure‐like events. The mice also showed hyperactivity and anxiety. Ricobaraza et al. (2019) suggested that the hypometabolism found in the human studies could be due to the influence of anti‐seizure drugs and differences in normalisation strategies used in the FDG‐PET assays. In human studies, FDG‐PET signals are usually presented as standardised uptake value (SUV) ratios, calculated as the SUV of the region of interest normalised to the SUV of a reference region, often the cerebellum or basal ganglia, that shows no difference in signal between the experimental groups being studied. The SUV is a measure of relative FDG uptake and is calculated as the radioactivity activity concentration divided by the FDG amount injected per gram body weight (Kinahan & Fletcher, 2010). Haginoya et al. (2018) and Kumar et al. (2018) used cerebellum and basal ganglia as reference regions, respectively, whereas Ricobaraza et al. (2019) used liver, since increased FDG uptake was found in the cerebellum and basal ganglia of their *Scn1a* ^WT/A1783V^ mice. However, these different normalisation strategies are unlikely to explain the opposing findings, as in all studies, the chosen reference regions showed no difference between Dravet versus control groups.

### 3.3 Glucose transporter abundance and levels of glucose and hexose‐phosphates in hippocampus

The findings from Ricobaraza et al. (2019) of increased brain FDG‐PET signals and interictal abnormalities in their *Scn1a* ^WT/A1783V^ mice raise further questions about alterations in glucose uptake and metabolism. Miljanovic, Hauck, et al. (2021); Miljanovic, van Dijk, et al. (2021) conducted proteomic and metabolomic studies, however, did not obtain or show data from FDG‐PET or EEG before sacrifice. Thus, it is difficult to reconcile data from the two laboratories. However, Miljanovic, van Dijk, et al. (2021) reasoned that due to increased amounts of hexose‐phosphates found, their data indicate increased glycolysis and glycogen breakdown in the *Scn1a* ^WT/A1783V^ mice, which is in line with the high FDG‐PET signals and interictal hyperactivity found by Ricobaraza et al. (2019).

Miljanovic, van Dijk, et al. (2021) identified a significant increase in hippocampal protein abundance of GLUT3 (+13%) in their 4‐week‐old *Scn1a* ^WT/A1783V^ mice (Miljanovic, van Dijk, et al., 2021), similar to studies in rats that found increased GLUT3 immunostaining signals after repeated kainic acid‐induced seizures (Gronlund et al., 1996) and increased *Slc2a3* (*Glut3*) mRNA expression after 1–4 hours of lithium pilocarpine‐induced status epilepticus (SE) (Leroy et al., 2011). Although the reasons for this are unclear, higher GLUT3 levels may be related to heightened interictal neuronal activity in the adult *Scn1a* ^WT/A1783V^ mice found in the earlier study and thus a presumably increased need for fuel (Ricobaraza et al., 2019). It is conceivable that an increase in GLUT3 abundance could facilitate the increased glucose utilisation seen in the FDG‐PET (Ricobaraza et al., 2019), but we note that even in *Glut3* hetero‐ or homozygous knockout mice with ~30–80% lower brain GLUT3 protein levels, no differences in whole brain ^3^H‐2‐deoxyglucose (^3^H‐2DG), ^14^C‐2DG or ^18^F‐FDG uptake were found (Shin et al., 2018; Stuart et al., 2011; Zhao et al., 2010). Miljanovic, van Dijk, et al. (2021) also discuss a 7% decreased protein abundance of glucose transporter 1 (GLUT1) in their *Scn1a* ^WT/A1783V^ mice (Miljanovic, Hauck, et al., 2021). While such decrease has potential to impede glucose uptake via the blood–brain barrier and/or into astrocytes and be in line with the findings from human FDG‐PET studies discussed above, this difference was not statistically significant (*p* = 0.06). Interestingly, there is only one study in humans that showed lower GLUT1 protein abundance in capillaries in brain tissue derived from epilepsy surgeries (Cornford et al., 1998). Surprisingly, there are no studies on neuronal or glial protein expression of the functional glucose transporter isoforms 1–4 in human epilepsies or chronic epilepsy models to our knowledge, despite the fact that glucose is needed to fuel learning and memory (e.g. Messier (2004), Mergenthaler et al. (2013)). In our opinion, this is an important basic knowledge gap to be filled.

Miljanovic, van Dijk, et al. (2021) found reduced glucose levels (~30%) but increased amounts of hexose‐phosphates and glycolytic metabolites in hippocampus (~30–400%). The interpretation of these data is unfortunately difficult, due to the reporting of relative metabolite amounts only, and the methods of euthanasia with anaesthesia (which slows glucose metabolism) and tissue collection used, which allows degradation of labile metabolites and increases lactate formation (see Section 6; Dienel, 2021). Plasma glucose levels were unchanged, so the reduced relative hippocampal glucose amounts are not due to hypoglycaemia. The lower brain glucose levels found in the *Scn1a* ^WT/A1783V^ mice could theoretically be explained by the increased amounts of glycolytic intermediates found. Interestingly, when assuming that normal levels of G6P in the mouse brain are around ~20–85 μM (Dienel, 2021; McDonald et al., 2017), then a doubling of G6P amounts as seen in Miljanovic, van Dijk, et al. (2021) could reduce hexokinase activity based on the *K*_i_ for G6P being 20 μM for hexokinase 1 and 2 (Wilson, 2003). Lower glucose levels were also identified in the *scn1Lab* larval zebrafish model of Dravet syndrome (Banerji et al., 2021), albeit in pooled whole larvae, not specifically in brain. Other potential explanations for reduced glucose levels are decreased vascularisation and/or cerebral blood flow, which remain to be examined in *Scn1a* ^WT/A1783V^ mice. Thus, further studies that combine FDG‐PET with ^13^C‐glucose tracking and EEG to assess glucose flux are needed to better understand changes in glucose utilisation in people with and in animal models of Dravet syndrome.

### 3.4 Glycolysis

Miljanovic, van Dijk, et al. (2021) reported increased levels of key glycolytic intermediates, including G6P, F6P, F16BP and pyruvate (~30–400%), in the hippocampus of adult *Scn1a* ^WT/A1783V^ mice, while hippocampal lactate levels were reduced (~20%) (Miljanovic, van Dijk, et al., 2021). Changes in protein abundance of glycolytic enzymes were also reported in hippocampal tissue in this model in young mice (Miljanovic, van Dijk, et al., 2021), although most changes were subtle, with 4–9% increased hexokinase 1 (HK1), glucose‐6‐phosphate isomerase (GPI) and pyruvate dehydrogenase E1 subunit alpha 1 (PDHA1), and 9% decreased L‐lactate dehydrogenase B (LDHB). While in support of the proposed increase in glycolysis, differences in total protein amounts of enzymes are difficult to interpret in isolation, as enzyme activities are largely regulated by post‐translational modifications and levels of substrates, products, cofactors as well as other key metabolites. Moreover, these changes in enzyme abundances are difficult to interpret in the context of the altered metabolite levels discussed above due to differences in the ages of the mice, with young 4‐week‐old versus adult ~22‐week‐old mice being used in the two studies. The authors interpreted their data to indicate that glycolysis is enhanced in their *Scn1a* ^WT/A1783V^ mouse model of Dravet syndrome, which is possible, and would be consistent with the increased FDG‐PET signals and interictal hyperactivity in brain reported by Ricobaraza et al. (2019) in adult *Scn1a* ^WT/A1783V^ mice in a different background strain. In addition, glycogen breakdown could lead to increased glycolytic metabolite levels (Section 3.5). On the other hand, increased glycolysis and glycogen breakdown during brain activation typically results in increased levels of lactate (Dienel, 2019a), whereas decreased lactate levels were reported, which raises further questions.

Reductions in the basal extracellular acidification rate (ECAR) and the mRNA expression of glycolytic enzymes have been reported in *scn1Lab* larval zebrafish (Kumar et al., 2016). Extracellular acidification of the assay buffer by whole live zebrafish larva was reduced at baseline in *scn1Lab* mutants, which displayed about 1 seizure‐like event per minute. Eight minutes after application of the voltage‐gated potassium blocker 4‐aminopyridine (4‐AP), wild‐type larvae showed severe seizure behaviour in about half of the larvae, characterised by frantic movement and an ECAR that increased by 3.6‐fold compared to baseline. This increased ECAR was maintained for the 48 min duration of the experiment, when at 48 min all larvae showed seizure behaviour, although less severe than at 8 min. The changes in ECAR in response to 4‐AP were blunted in *scn1Lab* mutant larvae, where ECAR increased more slowly and reached a 3.5‐fold relative to baseline maximum at about 30 min which was also maintained. This was interpreted by the authors to mean that during seizures, *scn1Lab* mutant larvae have a similar glycolytic capacity to wild‐type larvae (Kumar et al., 2016). Under basal conditions, *scn1Lab* mutants also exhibited about half of the mRNA levels of the glucose metabolism genes glucose‐6‐phosphatase catalytic subunit 1a (*g6pca.1)*, phosphoenolpyruvate carboxykinase 1 (*pck1*) and 2 (*pck2*), pyruvate dehydrogenase kinase isoform 2 (*pdk2)* and phosphorylase kinase catalytic subunit gamma 1 (*phkg1a)* compared to wild‐type larvae. Such changes might explain the low basal ECAR interpreted as low glycolytic activity in this model. In addition, a follow‐up paper from the same group reported about half of the total glucose amounts in the Dravet larvae as compared to wild‐type larvae (Banerji et al., 2021). Low glucose levels can be interpreted as lower or higher glucose utilisation, as total glucose levels in fish larvae can be regulated by the rates of glucose uptake, glycolysis as well as gluconeogenesis (discussed in Banerji et al., 2021). However, a low glycolytic rate in Dravet zebrafish larvae is also supported by the later study from this laboratory, which showed that compounds that increase phosphoenolpyruvate carboxykinase 1 mRNA, designed to increase gluconeogenesis, rescued the low glucose levels, glycolytic rate and mitochondrial respiration (see below), and reduced convulsive seizure‐like swim behaviour and the frequency of ictal‐ and interictal‐like forebrain electrographic events (Banerji et al., 2021). Interestingly, 1 hour after stimulation with 4‐AP, *pdk2* mRNA levels increased 15‐fold in *scn1Lab* mutants from baseline, compared to only twofold from baseline in wild‐type (Kumar et al., 2016), which might explain how Dravet larvae increased ECAR when stimulated with 4‐AP despite their lower ECAR at baseline. Please note that the sole use of the ECAR to assess the rate of glycolysis in whole live zebrafish larvae, without sequential addition of inhibitors of glycolysis, raises questions as to the specificity of these measurements. Additional uncontrolled factors that are expected to contribute to the acidification of the assay media include cutaneous gas exchange (i.e. the release of carbon dioxide, which may differ between genotypes) and the occurrence of seizures, which differ in frequency with treatment. Thus, application of 2DG is usually incorporated into the final part of the assay to control for the non‐glycolytic portion of the ECAR, but this glycolytic inhibitor may be toxic to whole zebrafish larvae. However, given that restoring glucose levels and the baseline ECAR with drugs that upregulate *pck1* mRNA was associated with a reduction in ictal and interictal electrographic activity, this suggests that changes in glucose metabolism are likely contributing to seizures in this model (Banerji et al., 2021). It is interesting to note that PCK2, PDK1 and PDK2 amounts also differed in *Scn1a* ^WT/A1783V^ mice, albeit these changes were found at the protein level, were of a smaller magnitude (PCK2–11%) and/or were in the opposite direction (PDK1 + 14%, PDK2 + 13%) (Miljanovic, Hauck, et al., 2021). Although it is unclear how metabolic findings obtained at the whole zebrafish level correspond to metabolic changes found in brain in people with Dravet syndrome, the *scn1Lab* model has proven translational potential. Drug screening in the *scn1Lab* larval zebrafish model identified the serotonin reuptake inhibitor fenfluramine as an anti‐seizure drug candidate for Dravet syndrome (Dinday & Baraban, 2015), which has shown efficacy in phase III clinical trials in children and young adults (*n* = 119) (Lagae et al., 2019) and is now approved by the United States Food and Drug Administration for the treatment of seizures in this disorder. Several other promising drug candidates have also been identified using this model (Banerji et al. (2021), see references cited therein)).

### 3.5 Glycogen metabolism

Little is known about glycogen metabolism in Dravet syndrome. Miljanovic, van Dijk, et al. (2021) found ~20% higher levels of glucose‐1‐phosphate (G1P) in the hippocampus of their *Scn1a* ^WT/A1783V^ mice. G1P is derived from G6P and used for glycogen synthesis but is also a metabolite produced during glycogen breakdown. To our knowledge, this is the only study to touch on glycogen metabolism in this disorder. The higher G1P amounts were interpreted as enhanced glycogenolysis in the Dravet mice (Miljanovic, van Dijk, et al., 2021), which fits with the interictal hyperactivity described (Ricobaraza et al., 2019). However, with increased glycogenolysis and high brain activity, increased lactate levels would be expected (Dienel, 2019b), but reduced lactate levels were reported (Miljanovic, van Dijk, et al., 2021). High G1P amounts could also equally indicate the opposite, that is, increased glycogen synthesis, especially because anaesthesia was used to sacrifice the mice and brain glycogen levels increase during anaesthesia (Fan et al., 2020). To our knowledge, except for ^14^C‐ or ^13^C‐glucose metabolism studies, there is no current method to determine whether glycogen breakdown or synthesis was altered in Dravet syndrome in people and mouse models (e.g. Dienel et al. (2007), DiNuzzo (2013)). Quantification of brain glycogen levels in *Scn1a* ^WT/A1783V^ mice will provide some more insight into if and how glycogen metabolism is affected in this model, and are of interest given reports of high brain glycogen content in people and in mice with chronic epilepsy (Dalsgaard et al., 2007; Seo et al., 2022). This is important to clarify, because as mentioned earlier, changes to brain glycogen metabolism may influence FDG‐PET signals (Dienel et al., 2023), which were found to be lower in people with Dravet syndrome (Ferrie et al., 1996; Haginoya et al., 2018; Kumar et al., 2018) but higher in *Scn1a* ^WT/A1783V^ mice (Ricobaraza et al., 2019).

### 3.6 TCA cycle and oxidative phosphorylation

Deficits in oxidative glucose metabolism in brain are widely reported in people with epilepsy and in rodent models of chronic epilepsy (reviewed in McDonald et al. (2018)), but how oxidative phosphorylation is impacted in people with Dravet syndrome remains poorly understood. Although there is no information regarding changes to oxidative glucose metabolism in brain in people with this disorder, deficits in mitochondrial parameters have been reported in muscle biopsies (Bolszak et al., 2009; Craig et al., 2012) and in primary skin fibroblasts (Doccini et al., 2015) from patients with Dravet syndrome. Craig et al. (2012) reported reduced electron transport chain (ETC) complex activities in muscle biopsies from two children with Dravet syndrome with confirmed mutations in *SCN1A*. One patient exhibited a 79% reduction in complex IV activity compared to control activity values, while the other patient showed 84% lower activity of complex III and elevated plasma levels of alanine and lactate. Both patients met the criteria for mitochondrial disease as defined by having less than 20% of the control activity ratio for an ETC complex normalised to citrate synthase. Similarly, Doccini et al. (2015) found reduced complex III activities in primary skin fibroblasts cultured from three out of four patients with Dravet syndrome, compared to those from five healthy age‐ and sex‐matched donors. This was associated with reductions in the protein levels of RISP and TTC19, which are components of complex III. Other ETC complex activities and protein levels were similar, as was the activity of the TCA cycle enzyme aconitase (Doccini et al., 2015). Although these studies are intriguing, extrapolation of these findings to metabolism in brain is difficult.

The previously discussed studies in *Scn1a* ^WT/A1783V^ mice also point towards impairments in oxidative glucose metabolism, based on reduced total levels of TCA cycle intermediates and metabolites as well as altered protein expression of enzymes involved in the TCA cycle. Miljanovic, van Dijk, et al. (2021) reported reduced relative levels of citrate and malate (~20% and ~10%, respectively) as well as the TCA cycle‐derived amino acids glutamate, glutamine and aspartate in the hippocampus of *Scn1a* ^WT/A1783V^ mice. Glucose metabolism in the TCA cycle is required for the synthesis of these amino acids, as ^13^C‐carbons from injected ^13^C‐glucose are found to a large extent in glutamate, glutamine, and aspartate within 15 min (reviewed in Dienel (2019a)). Thus, the low levels of these amino acids are consistent with the lower citrate and malate levels found and potential defective oxidative glucose metabolism in hippocampus. Taken together, this suggests impaired anaplerosis (refilling of C4 and C5 TCA cycle metabolites), which would be expected to impair TCA cycling, including the abilities to oxidise acetyl‐CoA and to produce ATP via oxidative phosphorylation. Impaired anaplerosis could be addressed by administration of anaplerotic substances, such as triheptanoin (Section 5.5). Interestingly, this finding of reduced citrate and malate levels in the adult mice was accompanied by increased hippocampal protein levels of enzymes involved in the TCA cycle in the 4‐week‐old *Scn1a* ^WT/A1783V^ mice (Miljanovic, Hauck, et al., 2021). Namely, isocitrate dehydrogenase [NAD] subunit alpha (IDH3A) (+6%), 2‐oxoglutarate dehydrogenase (OGDH) (+5%), succinate‐CoA ligase subunit alpha (SUCLG1) (+10%) and beta (SUCLA2) (+6%), succinate dehydrogenase complex flavoprotein subunit A (SDHA) (+4%) and subunit B (SDHB) (+3%), and malate dehydrogenase 2 (MDH2) (+8%). As discussed earlier, these findings are difficult to interpret due to differences in mouse age, and the fact that enzyme activities are influenced not only by their abundance. To determine how these changes affect oxidative glucose metabolism in the *Scn1a* ^WT/A1783V^ mice, functional data are required, such as that obtained by tracking the metabolism of ^13^C‐glucose in glycolysis and the TCA cycle in vivo or in acutely isolated brain slices (see Section 6).

In addition to the lower amounts of the amino acids glutamate, glutamine and aspartate, lower GABA levels were found in the hippocampus of the *Scn1a* ^WT/A1783V^ mice, although the GABA to glutamate ratio remained higher (Miljanovic, van Dijk, et al., 2021), in addition to 14% lower levels of glutamine synthetase (GlnS) (Miljanovic, Hauck, et al., 2021). Please note that the total concentrations of glutamate, glutamine and aspartate are very high in brain tissue (even higher than glucose levels). Thus, while only small amounts of these amino acids are used in neurotransmission, they largely function as amino acids in brain, for example, intermediates for other amino acids and metabolites as well as building blocks for proteins. In addition, it has been proposed that, similar to cancer cells, the brain may use glutamate as a source of fuel (Dienel, 2013; McKenna, 2013). Interestingly, reduced hippocampal protein levels of GlnS have been reported in post‐mortem hippocampal tissue collected from people with temporal lobe epilepsy (Eid et al., 2004; van der Hel et al., 2005) and in astrocytes in people with mitochondrial epilepsy (Chan et al., 2019). However, in epileptic human neocortex, GlnS activity was unchanged (Steffens et al., 2005). Both lower glutamate amounts and lower GlnS protein abundance may contribute to the reduced glutamine levels found in hippocampus (Miljanovic, van Dijk, et al., 2021), and glutamine–glutamine cycling was proposed to be impaired. As astrocytes harbour high amounts of glutamine and citrate as well as most of GlnS in brain (Sonnewald & Rae, 2010), these data also point towards alterations in astrocytes, which remain to be explored. In addition, altered abundances of proteins involved in glutamatergic and GABAergic signalling were found, which may contribute to the seizure generation and/or altered behaviour.

Similar to the findings in mice which indicate reduced ability to produce ATP in mitochondria, there is also evidence of reduced mitochondrial respiration in *scn1Lab* larval zebrafish in an extracellular flux analyser (Banerji et al., 2021; Kumar et al., 2016). While the first study reported reduced overall respiration, the later study found that maximal respiration, proton leak and spare respiratory capacity were lower in *scn1Lab* mutants compared to in wild‐type, while non‐mitochondrial respiration was unchanged (Banerji et al., 2021). In addition, while application of 4‐AP had no effect on the oxygen consumption rate (OCR) in wild‐type larvae, in *scn1Lab* mutants, 4‐AP triggered a gradual 42% increase in OCR over 30 min (Kumar et al., 2016). The meaning of this is unclear. No changes were found in mitochondrial DNA content (a crude marker of mitochondrial copy number), electron transport chain complex activities (I, II, III or IV), or activities of the TCA cycle enzymes aconitase, fumarase and malate dehydrogenase (Kumar et al., 2016). Given the decreased mRNA expression of *pdk2* reported in this zebrafish model (Kumar et al., 2016), it would be of interest to measure PDH activity, which may be higher in *scn1Lab* mutants if the low *Pdk2* mRNA results in lower protein levels and PDK2 is not otherwise activated.

### 3.7 Summary and conclusions

**FIGURE 2:**
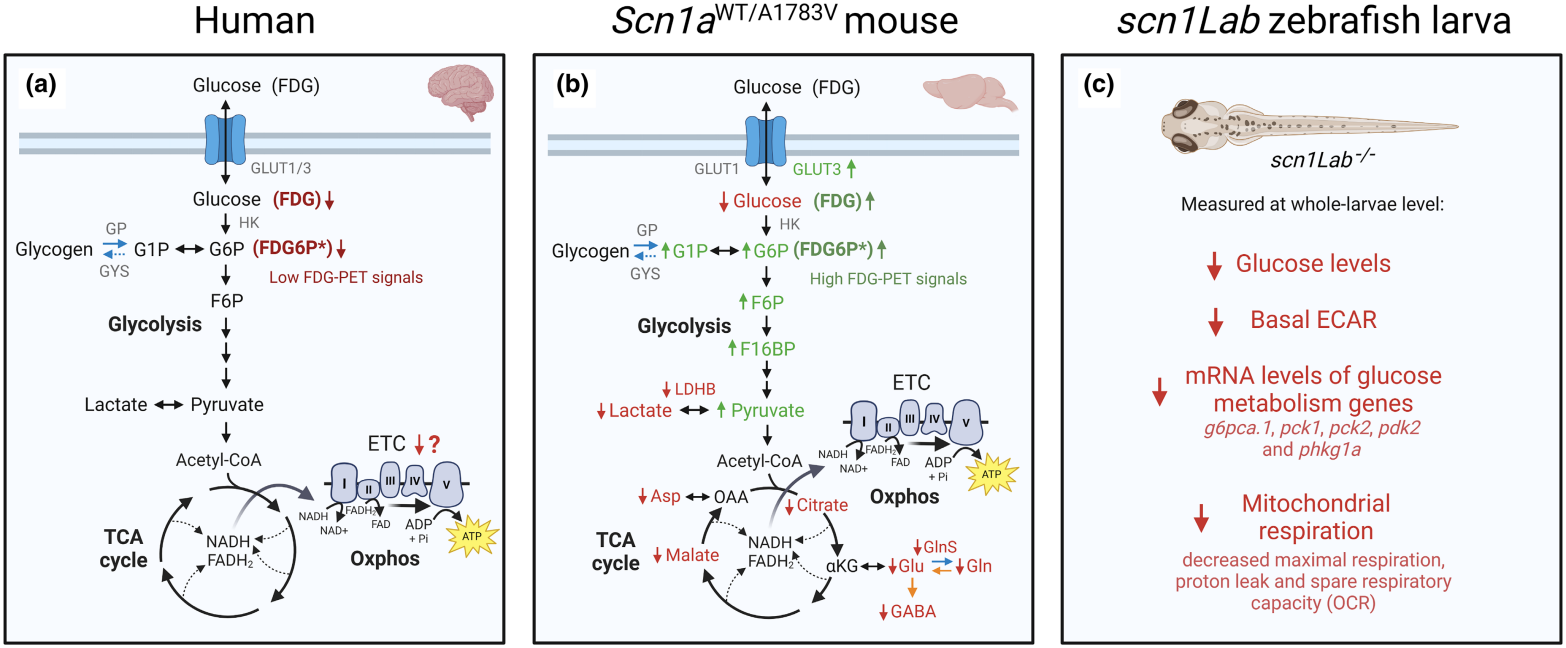
Summary of major metabolic changes found in brain in people with Dravet syndrome and in animal models thereof. (a) Interictal ^18^F‐fluorodeoxyglucose position emission tomography (FDG‐PET) signals are found to be low in people with Dravet syndrome (Ferrie et al., 1996; Haginoya et al., 2018; Kumar et al., 2018). Bold asterisk (*) indicates that FDG is phosphorylated by hexokinase (HK) to produce ^18^F‐fluorodeoxyglucose‐6‐phosphate (FDG6P), which is trapped in the cytosol. Electron transport chain (ETC) deficits were found in muscle biopsies and in cultured skin fibroblasts in Dravet syndrome patients (Bolszak et al., 2009; Craig et al., 2012; Doccini et al., 2015), but it is unclear if this occurs in brain (denoted by “?”). (b) Higher FDG‐PET signals were reported in *Scn1a* ^WT/A1783V^ mice (Ricobaraza et al., 2019). Metabolites measured by Miljanovic, van Dijk, et al. (2021) reflect relative hippocampal metabolite levels in adult *Scn1a* ^WT/A1783V^ mice, whereas protein levels measured in Miljanovic, van Dijk, et al. (2021) were from 4‐week‐old *Scn1a* ^WT/A1783V^ mice. Blue arrows indicate processes that are mostly astrocytic, while orange arrows indicate processes that are mostly neuronal. (c) Findings from *scn1Lab* larval zebrafish, assessed in whole larvae (Banerji et al., 2021; Kumar et al., 2016). GLUT1, glucose transporter 1. GLUT3, glucose transporter 3. GP, glycogen phosphorylase. GYS, glycogen synthase. OAA, oxaloacetate. Asp, aspartate. NADH, reduced nicotinamide adenine dinucleotide. FADH_2_, reduced flavin adenine dinucleotide. Oxphos, oxidative phosphorylation. ETC, electron transport chain. ADP, adenosine diphosphate; ATP, adenosine triphosphate; ECAR, extracellular acidification rate; *g6pca.1*, glucose‐6‐phosphate catalytic subunit 1A; OCR, oxygen consumption rate; *pck1*, phosphoenolpyruvate carboxykinase 1; *pck2*, phosphoenolpyruvate carboxykinase 2; *pdk2*, pyruvate dehydrogenase kinase 2; *phkg1a*, phosphorylase gamma kinase 1a; Pi, inorganic phosphate.

Metabolic changes are apparent in people with Dravet syndrome and in animal models of this disorder (summarised in Figure 2). Differences in experimental design and a discordance in findings between human, mice and zebrafish studies render determination of an overall metabolic picture difficult at this time. Despite small sample sizes, findings from FDG‐PET studies in humans show that interictal FDG‐PET signals in people with Dravet syndrome above age 3 are low (Ferrie et al., 1996; Haginoya et al., 2018; Kumar et al., 2018). However, the utility of this data with regard to defining specific metabolic changes occurring in brain in Dravet syndrome is limited, as low FDG‐PET signals only indicate reduced glucose uptake and/or hexokinase activity, and can be influenced by changes in metabolism that are rarely considered, such as altered glycogen utilisation in brain (Dienel et al., 2023). These findings in humans appear to align with findings from zebrafish larvae, which point towards an overall glucose hypometabolism, with reduced expression of glucose metabolism genes and low rates of extracellular acidification and mitochondrial respiration (Banerji et al., 2021; Kumar et al., 2016). The reduced levels of citrate, malate, glutamate, glutamine and aspartate in the Dravet mice also indicate reduced oxidative glucose metabolism. These data are generally in line with what is reported in people with temporal lobe epilepsy and in chronic rodent epilepsy models, namely, reductions in brain metabolism of glucose, TCA cycle activity and/or oxidative phosphorylation (reviewed in McDonald et al. (2018) and Dienel et al. (2023)). This is expected to lead to reduced energy levels, which especially during brain stimulation and stress, conditions that are well known to increase susceptibility to seizures, may lead to impairments in ion homeostasis and therefore contribute to the generation of seizures (see Section 2.1). Thus, from a biochemical perspective, the ketogenic diet, which provides auxiliary fuels in the form of βHB and acetoacetate in addition to glucose among many other proposed actions, is a reasonable anticonvulsant approach.

Studies in *Scn1a* ^WT/A1783V^ mice partially showed the opposite from studies in human and zebrafish, with high FDG‐PET signals reported (Ricobaraza et al., 2019) and metabolomic and proteomic studies pointing towards increased glycolysis (Miljanovic, Hauck, et al., 2021; Miljanovic, van Dijk, et al., 2021). These results may be due to neuronal hyperactivity, which is well known to increase glucose utilisation and glycolysis (Dienel, 2019a). Functional assays such as ^13^C‐glucose metabolism tracking ideally somehow paired with electrophysiological measurements are required to confirm increased glucose transport and glycolysis in the *Scn1a* ^WT/A1783V^ mouse model (see Section 6).

## 4 METABOLIC ASPECTS OF *KCNA1* EPILEPSY

### 4.1 *KCNA1* epilepsy

**FIGURE 3:**
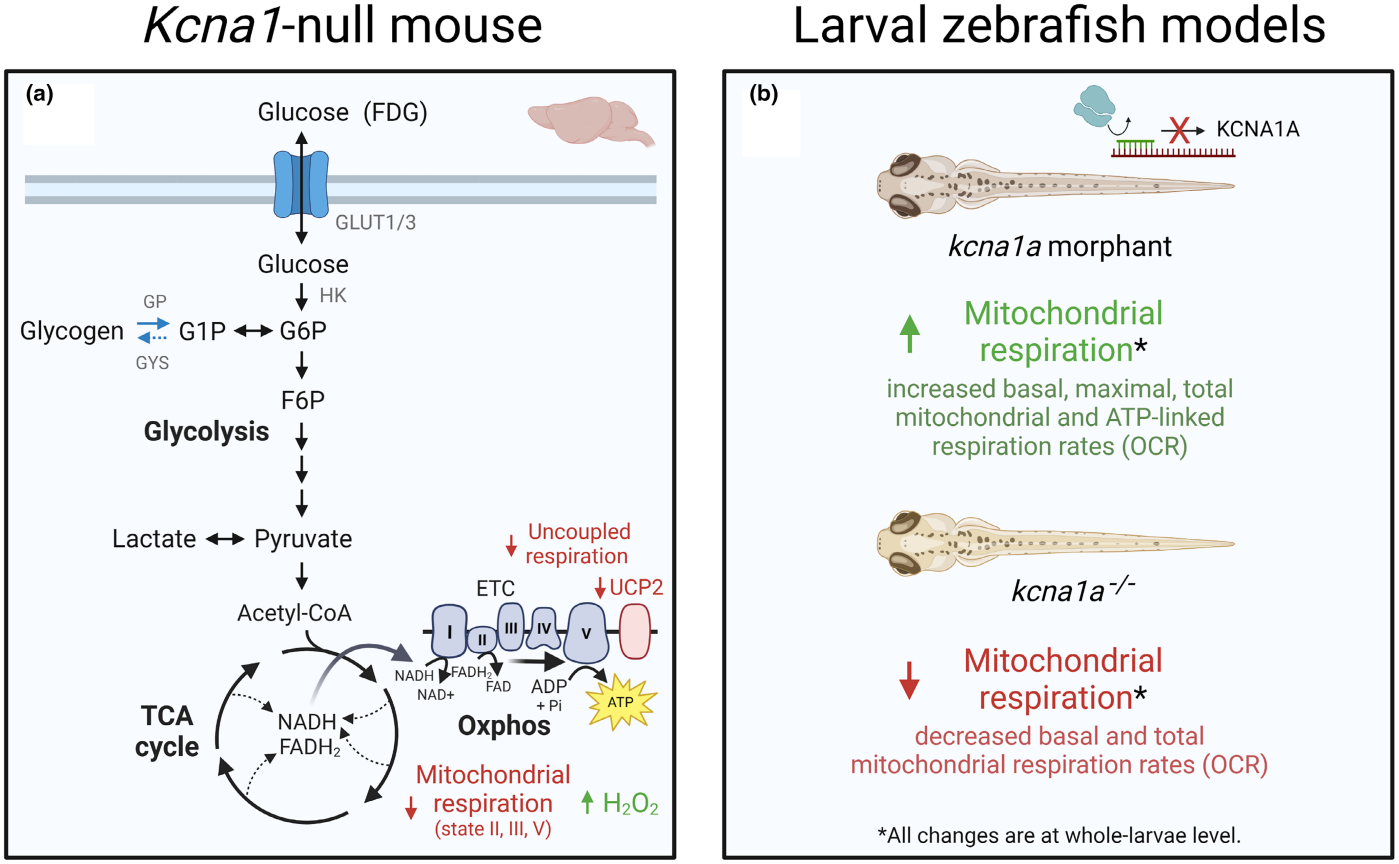
Summary of major metabolic changes in animal models of *KCNA1*‐associated epilepsy. (a) Metabolic alterations in brain in the *Kcna1* ^−/−^ (*Kcna1*‐null) mouse model (Roundtree et al., 2016; Simeone et al., 2014). (b) Metabolic alterations in two zebrafish models of *kcna1a* deficiency, morpholino antisense knockdown and *Kcna1a* ^−/−^ mutants (Dogra et al., 2023; Ibhazehiebo et al., 2018; Stackley et al., 2011). Blue arrows indicate processes that are mostly astrocytic. ADP, adenosine diphosphate; ATP, adenosine triphosphate; ETC, electron transport chain; FADH_2_, reduced flavin adenine dinucleotide; GLUT1, glucose transporter 1; GLUT3, glucose transporter 3; GP, glycogen phosphorylase; GYS, glycogen synthase; NADH, reduced nicotinamide adenine dinucleotide; OCR, oxygen consumption rate; Oxphos, oxidative phosphorylation; Pi, inorganic phosphate; UCP2, mitochondrial uncoupling protein 2.

Mutations in the *KCNA1* gene, encoding for the alpha subunit of the voltage‐gated potassium channel Kv1.1, in humans can produce a broad spectrum of phenotypes but most commonly lead to development of episodic ataxia type 1 (EA1), a rare neurological movement disorder (Paulhus et al., 2020). Epilepsy is a frequently occurring comorbidity in people with EA1, but *KCNA1* mutations in some people also cause epilepsy without causing EA1. It has been suggested that the occurrence of epilepsy or seizures in people with *KCNA1* mutations may depend on where the mutation is located within the *KCNA1* gene (Paulhus et al., 2020). In the absence of available drugs opening *KCNA1* channels, people affected by *KCNA1* mutations are usually treated with the sodium channel blocker carbamazepine or the carbonic‐anhydrase inhibitor acetazolamide (Graves et al., 2014; Imbrici et al., 2016; Jen et al., 2007;Lauxmann et al., 2021; Orsucci et al., 2019). To our knowledge, ketogenic diet is not used. However, several studies have investigated brain metabolism, the effects of ketogenic diet and seizure generation in mice lacking this potassium channel. *Kcna1* ^*−/−*^ mice (*Kcna1*‐null) develop spontaneous recurrent seizures at around 3 weeks of age at a frequency of ~6–12 seizures per day (Chun et al., 2018; Fenoglio‐Simeone et al., 2009; Kim et al., 2015; Simeone et al., 2016; Simeone et al., 2021) and experience premature death (Chun et al., 2018; Simeone et al., 2016). The high mortality in *Kcna1*‐null mice appears to coincide with a decline in cardiorespiratory function (Aiba & Noebels, 2015; Glasscock et al., 2010; Simeone et al., 2018), making it an ideal model to study sudden unexplained death in epilepsy (SUDEP) (Manolis et al., 2019). Interestingly, these mice exhibited cell death in CA1 but not CA3 of the hippocampus (Simeone et al., 2021). *Kcna1a* (*kcna1a* and *kcna1b* are the zebrafish paralogues of the human *KCNA1*) knockdown and knockout zebrafish larvae have also been used as models of epilepsy (Dogra et al., 2023; Ibhazehiebo et al., 2018). Compared to Dravet syndrome, considerably less is known about how metabolism is affected in *KCNA1* epilepsy. To our knowledge, alterations in brain glucose metabolism have not been investigated in people with *KCNA1* epilepsy, which might be explained by the fact that loss of *KCNA1* function in humans can manifest in many phenotypes of which ataxia is dominant and cerebellar hypometabolism has been reported in one patient (Kim et al., 2008). The metabolic data that are available from mouse and zebrafish models are reviewed below and summarised in Figure 3.

### 4.2 Deficits in mitochondrial respiration in models of *KCNA1* epilepsy

Impaired mitochondrial respiration has been reported in adolescent *Kcna1*‐null mice (P32‐38). Simeone et al. (2014) found reduced state III and state V respiration rates in isolated mitochondria from both the cortex and hippocampus of *Kcna1*‐null mice compared to wild‐type. However, no differences in the protein abundance of complexes I‐V were found, as measured by Western blotting using antibodies specific to a representative protein from each electron transport chain complex. Mitochondrial H_2_O_2_ levels were increased in the cortex and hippocampus of the *Kcna1*‐null mice. Lower protein abundance of the mitochondrial uncoupling protein UCP2 and a reduced uncoupled respiration rate (in response to free fatty acids) in *Kcna1*‐null mitochondria were also found. Similar findings were reported in a more recent study by the same group, with mitochondria isolated from hypothalamic tissue of *Kcna1*‐null mice displaying reduced state II, III and V respiration rates compared to wild‐type mice (Roundtree et al., 2016).

Changes to mitochondrial respiration have also been identified in ‘epileptic’ *kcna1a* larval zebrafish models, although in different directions in two different larvae models. Ibhazehiebo et al. (2018) knocked down *kcna1a* using a morpholino antisense approach. Zebrafish embryos were injected with the morpholino at the single cell stage, which halved the mRNA levels of *kcna1a* at 3 days post‐fertilisation (dpf). *Kcna1a* morphants showed hyperactive locomotor behaviour at 5 dpf, and abnormal EEG activity measured via extracellular field recordings from the tectum was found at 5 and 6 dpf, indicative of seizure‐like activity. Knockdown of *kcna1a* was associated with increased basal, maximal, ATP‐linked and total mitochondrial respiration rates at 2 dpf in whole larvae placed in an extracellular flux analyser. Similar changes in mitochondrial respiration were seen in wild‐type larvae pre‐treated with PTZ for 10 min (Ibhazehiebo et al., 2018), which like the *kcna1a* morphants showed hyperexcitability and increased locomotion (Afrikanova et al., 2013; Baraban et al., 2005). The higher rates of mitochondrial respiration found in the *kcna1a* morphants are mostly likely due to respiration being measured during periods of high neuronal activity, as anticonvulsants reduced the enhanced respiration (Ibhazehiebo et al., 2018). Curiously, the anticonvulsants did not lower the high locomotor activity, although they decreased respiration, which indicated that they reduced brain (hyper‐)activity.

In a recent preprint, a new *kcna1a* (*kcna1a* ^−/−^) mutant zebrafish model is described (Dogra et al., 2023). The *kcna1a* mutant larvae, similar to the morphants, displayed locomotor deficits, uncoordinated movements and similar seizure‐like activity, as well as altered head mRNA expression of v‐fos FBJ murine osteosarcoma viral oncogene homologue Ab (*fosab*, orthologous to human *FOS* / c‐Fos) and the vesicular glutamate transporter *vglut2a* (both increased) and glutamate decarboxylase 1b (*gad1b*) (decreased) at 3 and 5 dpf. However, opposite to the morphants, *kcna1a* mutants displayed decreased rates of mitochondrial respiration at 3 and 6 dpf in whole larvae. At 3 dpf, basal and non‐mitochondrial respiration were lower, while at 6 dpf, basal and total mitochondrial respiration were reduced (note that the effects on non‐mitochondrial respiration should be interpreted with caution, see Divakaruni and Jastroch (2022)). This difference in findings might be attributed to the different methods employed to knockdown *kcna1a*, as multiple studies have shown poor agreement between morphant and mutant phenotypes (Kok et al., 2015; Lawson, 2016). Morpholino antisense oligonucleotides can produce off‐target effects, of which induction of apoptosis via activation of p53 is common (Eisen & Smith, 2008; Kok et al., 2015; Lawson, 2016). Although off‐target effects on p53 were controlled for with a second morpholino targeting p53 in Ibhazehiebo et al. (2018), the adequacy of this control has been questioned (Kok et al., 2015), and it would not control for any off‐target effects independent of p53. Genetic compensation in *kcna1a* mutants but not morphants is another possibility (Morcos et al., 2015; Salanga & Salanga, 2021).

### 4.3 Summary and conclusions

Mitochondrial respiration is seemingly reduced in animal models of *KCNA1* loss of function, based on data from mice lacking *Kcna1* and from genetic *kcna1a* mutant zebrafish larvae. However, there is no information about how other metabolic pathways, like glycolysis or the TCA cycle, are impacted. While rodents have been classically used in studies of brain metabolism, zebrafish models are becoming increasingly common, with many models for specific channelopathies available (Kolesnikova et al., 2022). It is interesting to note that the sodium channel blocker carbamazepine, which seems to be the current treatment of choice for EA1 (Lauxmann et al., 2021; Orsucci et al., 2019) despite no proven efficacy in clinical trials (D'Adamo et al., 2015), was reported to have efficacy in *kcna1a* ^−/−^ larval zebrafish (Dogra et al., 2023), but only partial or no efficacy in *Kcna1*‐null mice (Deodhar et al., 2021; Dogra et al., 2023). The reasons for this remain unclear, but this finding showcases the value of zebrafish models in the study of specific genetic epilepsies.

## 5 DIETARY METABOLIC THERAPIES FOR GENETIC ION CHANNEL EPILEPSIES

### 5.1 A case for dietary metabolic therapies in certain genetic ion channel epilepsies

Some of the metabolic changes discussed above render these genetic ion channel epilepsies amenable to treatment with dietary metabolic therapies, including ketogenic diet, medium‐chain triglycerides or triheptanoin. Via the provision of auxiliary brain fuels such as ketone bodies and/or MCFAs or anaplerotic fuels, these treatments could address some of the metabolic deficits that are likely to be contributing to seizure generation, although the mechanisms through which these elicit their anticonvulsant action are debated (see Section 2.1). The effects of these dietary metabolic therapies in people with and animal models of Dravet syndrome and *KCNA1* epilepsy are reviewed below.

### 5.2 Ketogenic diet in Dravet syndrome

A recent meta‐analysis of seven published trials on the use of an add‐on ketogenic diet in people with Dravet syndrome (of which 80–100% had an *SCN1A* mutation) revealed good efficacy of this treatment (Wang et al., 2020). Pooled efficacy rates for ≥50% seizure reductions were 63%, 60% and 47% at 3, 6 and 12 months after ketogenic diet commencement (3:1–4:1 ratios of fat to protein plus carbohydrates used), respectively, and ~14% (*n* = 23 of 167) of all patients from the included studies were seizure‐free at 12 months (Wang et al., 2020). These efficacies are comparable to those found in other studies of the ketogenic diet for drug‐resistant epilepsies (Liu et al., 2018) and to that reported in a recent meta‐analysis of fenfluramine trials in people with Dravet syndrome (Sharawat et al., 2021). In Wang et al. (2020), pooled efficacy rates decreased with time, potentially due to poorer compliance. This is in line with decreased pooled retention rates at 6 and 12 months of 78% and 49%, respectively, potentially indicating that people with Dravet syndrome find this ketogenic diet difficult to follow. Interestingly, a retrospective study of ketogenic diet treatment showed that individuals with mutations in *SCN1A* had a higher responder rate of 78% (*n* = 14 of 18, with ≥90% reductions in seizure frequency from baseline) at 3, 6 and 12 months, compared to people without identified mutations, who had responder rates of about 30% at those times (*n* = 24–27 of 82) (Ko et al., 2018).

Beneficial effects of the ketogenic diet are also reported in animal models of Dravet syndrome (Table 1). In both wild‐type and *Scn1a* ^*+/−*^ heterozygous knockout mice, 14 days of consuming a 6:1 ketogenic diet initiated at weaning on post‐natal day 21 increased seizure thresholds in a flurothyl (a GABA_A_ receptor blocker) seizure test, indicated by an increased latency to tonic hindlimb extension (Dutton et al., 2011). In both standard diet‐ and ketogenic diet‐fed *Scn1a* ^*+/−*^ mice, seizure thresholds were lower compared to the wild‐type mice fed the equivalent diets. However, ketogenic diet feeding virtually restored the seizure thresholds of *Scn1a* ^*+/−*^ mice to the levels seen in wild‐type mice fed a standard diet. Note that in rodent studies, ketogenic diets often contain a higher fat content (6:1–6.3:1) as compared to those consumed by humans (3:1–4:1), potentially because ketone body levels are relatively lower in adolescent rodents when fed ad libitum ketogenic diets (around 1–2.5 mM) (Bough et al., 2002; Samala et al., 2008) compared to humans (1–5 mM) (Rho & Boison, 2022), and that anticonvulsant effects appear to be more consistently found with the higher ratio in acute mouse models (Borges, 2008). Similar protective effects were seen in ketogenic diet‐fed *Scn1a* ^RH/+^ mice (Dutton et al., 2011), which are knock in mice with the human *SCN1A* GEFS+ mutation, R1648H (Martin et al., 2010). In *Scn1a* ^R1407X/+^ mice, a 6.3:1 ketogenic diet initiated at weaning improved mortality rate, with survival at 60 days increasing from 44% in standard diet‐fed mice to 86% with ketogenic diet (Teran et al., 2019). *Scn1a* ^R1407X/+^ mice experienced on average two spontaneous seizures per day, which increased in frequency 1–2 days prior to death. However, the lower mortality in the *Scn1a* ^R1407X/+^ mice‐fed ketogenic diet was not associated with decreased behavioural seizure frequency (Teran et al., 2019). The authors suggested that this protective effect could alternatively be explained by the ketogenic diet preventing seizure propagation from the forebrain to brainstem nuclei that are important for cardiorespiratory control (thus preventing SUDEP) and/or a protection against cytological changes such as gliosis and cell death (which they did not explore). We agree that these are reasonable possibilities, especially since it has been demonstrated that the ketogenic diet protected against cell death in *Kcna1*‐null mice (Simeone et al., 2021), but we note that in these mice, the ketogenic diet has very clear anti‐seizure effects (Table 2). An important limitation of Teran et al. (2019) is that only convulsive motor seizures were assessed (by video review by a blinded observer) and no EEG recording was used. Thus, another possibility could be that in these mice, ketogenic diet may be reducing the frequency of non‐convulsive seizures and/or interictal abnormal activity. This may have reduced abnormal activity in brain areas that control cardiorespiratory functions and cause SUDEP. This possibility is supported by the characteristics of the *Scn1a* ^WT/A1783V^ mice, which at room temperature did not exhibit behavioural seizures but showed interictal epileptiform discharges (Ricobaraza et al., 2019). However, the effects of ketogenic diet were not tested in those Dravet mice. Lower mortality was also found in a more recent study by the same group, where survival of *Scn1a* ^R1407X/+^ mice at 60 d increased from 61% to 92% with ketogenic diet feeding (Crotts et al., 2021). In this study, a third group of *Scn1a* ^R1407X/+^ mice was treated with the ketogenic diet plus 5% glucose (w/v) in the drinking water. This prevented ketosis in these mice. This was interpreted by the authors to mean that ketosis was not required for the lifespan‐extending effects of the ketogenic diet in *Scn1a* ^R1407X/+^ mice (Crotts et al., 2021). Indeed, whether the anticonvulsant effects of the ketogenic diet are mediated by the sole direct effects of ketone bodies is debated, as low glucose blood levels or low caloric intake may play a role (Rho & Boison, 2022). In *Scn1a* ^WT/A1783V^ mice, 3 weeks of ketogenic diet feeding did not affect motor seizure frequency, duration or severity (measured with video‐EEG‐ECG), frequencies of interictal spiking or spike–wave discharges, or background activity on the EEG (Miljanovic, van Dijk, et al., 2021). In Miljanovic, van Dijk, et al. (2021), the ketogenic diet was started when mice were 16 weeks old, whereas in the other three studies, it was initiated earlier at weaning (Crotts et al., 2021; Dutton et al., 2011; Teran et al., 2019), and mortality rates were not assessed. However, despite lack of anticonvulsant effects, some metabolic effects of the ketogenic diet were identified, most notably, increased relative hippocampal amounts of G6P, DHAP and 6‐phosphogluconate (6PG) as compared to standard‐fed *Scn1a* ^WT/A1783V^ mice (Miljanovic, van Dijk, et al., 2021). In the authors' proteomic study, 4‐week‐old standard diet‐fed *Scn1a* ^WT/A1783V^ mice showed increased hippocampal levels of ketone body transport and metabolism proteins compared to standard diet‐fed wild‐type mice (Miljanovic, Hauck, et al., 2021). Namely, monocarboxylate transporter 1 (MCT1) levels were 20% increased, and 3‐hydroxy‐3‐methylglutaryl‐CoA synthase‐2 (HMGCS2) levels were 24% increased. Subtle increases in the protein levels of succinyl‐CoA:3‐ketoacid CoA transferase (SCOT, +4%) and acetyl‐CoA acetyltransferase 1 (ACAT1, +7%) were also found. In brain, MCT1 is expressed predominantly in endothelial cells and astrocytes and mediates uptake of acetoacetate and βHB (as well as lactate and pyruvate). Ketone body metabolism in brain appears to be limited mostly by uptake at the blood–brain barrier, as the *K*_m_ of the endothelial and astrocytic MCT1 for βHB is 12.5 mM (Enerson & Drewes, 2003), while non‐pathological blood βHB levels are below 5 mM, even while on a ketogenic diet. The lower *K*_m_ of 1.2 mM of the neuronal MCT2 transporter is likely responsible for the largely neuronal metabolism of ketone bodies (Enerson & Drewes, 2003); however, it may still limit metabolism as extracellular fluid βHB concentrations were only 50 μM in mice fed a ketogenic diet (Samala et al., 2011). Thus, the 20% increased MCT1 levels are likely to facilitate increased ketone body uptake in brain in *Scn1a* ^WT/A1783V^ mice. The effects of 24% increased HMGCS2 are less clear, as this enzyme is involved in ketogenesis, not ketolysis. A possible explanation for increased levels of the ketone body metabolism enzymes and the ketone body transporter MCT1 is developmental delay in the Dravet mice, as seen in humans with Dravet syndrome (Dravet, 2011), since brain ketone body utilisation and the expression of these proteins are high in rats and mice in early post‐natal life and decrease with age (Düking et al., 2022; Nehlig, 1999; Nehlig & Pereira de Vasconcelos, 1993).

**TABLE 1:** Effects of the ketogenic diet in animal models of Dravet syndrome.

Reference	Species	Model	Diet ratio of fat vs. non‐fat^a^ / composition, duration and age when started	Effects of ketogenic diet (KD) on	
				Seizures and mortality	Metabolism
Dutton et al. (2011)	Mouse	*Scn1a* ^+/−^ mice *Scn1a* ^R1648H /+^ mice^b^	6:1 14 days started at P22	**↑ seizure thresholds** in acute flurothyl seizure test in both *Scn1a* ^+/−^ and *Scn1a* ^*RH/+*^ mice, to levels close to SD‐fed wild‐type mice	N.D.
Teran et al. (2019)	Mouse	*Scn1a* ^R1407X/+^ mice	6.3:1 from weaning to P60	↔ motor seizure frequency **↓ mortality** (14% mortality in KD‐fed vs 56% in SD‐fed)	N.D.
Crotts et al. (2021)	Mouse	*Scn1a* ^R1407X/+^ mice	6.3:1 from weaning to P60	**↓ mortality** (8% mortality in KD‐fed vs 39% in SD‐fed)	N.D.
Miljanovic, van Dijk, et al. (2021)	Mouse	*Scn1a* ^WT/A1783V^ mice	6:1 5 weeks started at P105	↔ motor seizure frequency, duration or severity ↔ frequency of interictal spikes ↔ EEG background activity	**↑ G6P**, **DHAP** and **6PG** levels in hippocampus
Baraban et al. (2013)	Zebrafish	*scn1Lab* ^−/−^ larval zebrafish	Palmitate, myristate, laurate and caprate (all 200 μM) and 100 μM phosphatidylcholine in embryo media. 48 h from 4 dpf	**↓ seizure‐like behaviour** in locomotion assay **↓ burst frequency** on forebrain extracellular field recordings	N.D.
Kumar et al. (2016)	Zebrafish	*scn1Lab* ^−/−^ larval zebrafish	200 μM palmitate, 200 μM laurate and 100 μM phosphatidylcholine in embryo media 48 h from 4 to 6 dpf	N.D.	**↑** basal **ECAR** and **OCR** to wild‐type level

Abbreviations: dpf, days post‐fertilisation; KD, ketogenic diet; N.D., no data; P, post‐natal day; SD, standard diet.

^a^ For rodent and human ketogenic diets, the ‘ketogenic ratio’ is provided, which refers to the ratio of fat to non‐fat (protein plus carbohydrates) in the diet measured by weight. For ketogenic diets in humans, ketogenic ratios of 4:1 and 3:1 are standard.

^b^ *Scn1a* ^*R1648H /+*^ mice are a model of generalised epilepsy with febrile seizures plus (GEFS+), all other models are referred to by the authors as models of Dravet syndrome.

**TABLE 2:** Effects of the ketogenic diet in *Kcna1*‐null mice.

Reference	Diet ratio of fat vs. non‐fat^a^ / composition, duration and age when started	Effects of ketogenic diet (KD) on seizures and mortality
Fenoglio‐Simeone et al. (2009)	6.3:1 4 weeks started at P18‐20	**↓ seizure frequency** (video‐EEG)
Kim et al. (2015)	6.3:1 3 weeks started at P18‐P20	**↓ seizure frequency** (video‐EEG, total seizures and tonic–clonic seizures)
Simeone et al. (2016)	6.3:1 Started at P21 until death/end of experiment	**↓ behavioural seizure frequency** (observed for 15 min x 8 times/day) ↓ **seizure severity** **↑ lifespan** 43 d in SD‐fed vs 63 d in KD‐fed
Chun et al. (2018)	6.3:1 Started at P25, P30 or P35 until death/end of experiment	**↓ behavioural seizure frequency** (continuous video PND40‐45). **↑ lifespan** from 47 d in SD‐fed to 70 d in KD‐fed
Simeone et al. (2021)	6.3:1 17 days started at P20	**↓ seizure frequency** (video‐EEG) **↓ cell death** in hippocampal CA1 area

Abbreviations: d, days; KD, ketogenic diet; P, post‐natal day; SD, standard diet.

^a^ For rodent and human ketogenic diets, the ‘ketogenic ratio’ is provided, which refers to the ratio of fat to non‐fat (protein plus carbohydrates) in the diet measured by weight.

In *scn1Lab* larval zebrafish, a “ketogenic diet” consisting of palmitate (C16:0), myristate (C14:0), laurate (C12:0), caprate (C10:0) and phosphatidylcholine added to the embryo media decreased seizure‐like behaviour in a locomotion assay and reduced burst frequency measured in field recordings (Baraban et al., 2013). Fatty acid treatment also increased the basal ECAR and OCR in these mutants (Kumar et al., 2016), which suggests that these improvements in phenotype may be in part mediated by improved energy metabolism.

### 5.3 Ketogenic diet in *KCNA1* epilepsy

The effects of ketogenic diet feeding in *Kcna1*‐null mice have been previously reviewed (Ren et al., 2019) and are summarised here in Table 2, which includes several more recent studies in mice as well as studies in zebrafish. Of particular relevance to SUDEP is that the ketogenic diet has clear effects on lifespan in *Knca1*‐null mice, increasing lifespan by 47–48% from standard diet‐fed *Kncna1*‐null mice (Chun et al., 2018; Simeone et al., 2016). This lifespan extension was more pronounced when the ketogenic diet was initiated earlier in life (Chun et al., 2018). While standard diet‐fed *Knca1*‐null mice died at similar ages irrespective of sex, ketogenic diet feeding increased survival in females to a greater extent than in males (+12%), which was associated with higher plasma βHB levels in females (Chun et al., 2018). Several studies also reported reduced video‐EEG and/or behavioural seizure frequencies in *Knca1*‐null mice (Chun et al., 2018; Fenoglio‐Simeone et al., 2009; Kim et al., 2015; Simeone et al., 2016; Simeone et al., 2021). Ketogenic diet feeding in *Kcna1*‐null mice also minimised cell death that occurs in the CA1 of the hippocampus in standard diet‐fed *Kcna1*‐null mice (Simeone et al., 2021).

### 5.4 Medium‐chain triglycerides

Due to issues with the palatability and strict nature of ketogenic diets, alternatives are needed.

There is increasing evidence that incorporation of medium‐chain triglycerides into a non‐ketogenic diet is effective in some people and in dogs with epilepsy, as well as in rodent seizure and chronic epilepsy models (Han, Conboy‐Schmidt, et al., 2021; Neal et al., 2022). Commercially available medium‐chain triglyceride oils usually contain a mixture of octanoic (C8) and decanoic (C10) acids (30:70–70:30), and sometimes dodecanoic acid (C12). In our opinion, the anticonvulsant effects of medium‐chain triglycerides are most likely attributable to the fact that they are metabolised into MCFAs, which are used as brain fuel (Andersen et al., 2021, 2023), but other additional mechanisms of action are being discussed, including ketone body generation and AMPA receptor inhibition by decanoic acid (summarised by Han, Conboy‐Schmidt, et al. (2021)). To our knowledge, there are no trials of add‐on medium‐chain triglycerides in people with specific genetic ion channel epilepsies. In a recent open‐label feasibility study of K.Vita, a drink that contains medium‐chain triglycerides with a 80:20 mix of decanoate and octanoate and provided up to 35% of daily calories, 38% of children (*n* = 6 of 16) experienced ≥50% reductions in the frequency of seizures or paroxysmal events (Schoeler et al., 2021). All children in the study had early‐onset epilepsy due to either confirmed or presumed genetic mutations, 23% of which had Dravet syndrome (*n* = 8 of 35). Based on the paper, it is not clear whether they responded to the treatment, as this was not a goal of the study. A recent study in *Scn1a* ^R1407X/+^ mice tested the effects of diets containing medium‐chain triglycerides with either pure decanoate or different combinations of octanoate and decanoate (20:80 C8/C10 and 60:40 C8/C10) at 35% of total daily calories (Jancovski et al., 2021). These Dravet mice fed standard chow experienced spontaneous seizures, which started at P16 (~0.5 seizures/day) and peaked at P23 (~2 seizures/day), before returning to earlier levels by P26. Mice showed high early mortality beginning at ~P20, with death preceded by convulsive seizures in all mice. At P18, mice were switched to one of the three medium‐chain triglyceride diets or maintained on standard chow. All three experimental diets extended survival in the Dravet mice, with 80–82% survival at P46, compared to only 50% survival in mice fed standard chow. All three experimental diets delayed the time to onset of hyperthermia‐induced seizures compared to the chow diet, but only diets with both octanoate and decanoate reduced spontaneous convulsive seizure frequencies at P22–P25. Various markers of mitochondrial function and antioxidant defence were also increased in whole brain but did not correlate with the fed MCFA amounts.

We also recently showed that the MCFAs octanoic and decanoic acid are metabolised in a different cell type (astrocytes) compared to βHB (neurons) in acute mouse cerebral cortex slices, with little competition found between these substrates (Andersen et al., 2023). Moreover, in the K.Vita study, the anticonvulsant effects found in children and adults correlated with increased levels of MCFAs and not with levels of βHB, which remained low (Schoeler et al., 2021). Thus, incorporation of medium‐chain triglycerides into ketogenic or modified Atkin's diets that keep βHB levels high theoretically may optimise fuel delivery to both major cell types in brain (Andersen et al., 2023). This may be particularly beneficial in people with genetic epilepsies that already respond well to these high‐fat diets and should be tested.

### 5.5 Triheptanoin

The finding that the levels of citrate and malate and several amino acids derived from the TCA cycle were reduced in Dravet mice (Miljanovic, van Dijk, et al., 2021) suggests impaired anaplerosis, which could be addressed by adding triheptanoin (the triglyceride of heptanoic acid) to the diet. Triheptanoin was found to be anticonvulsant in several acute and chronic seizure models and appeared to be safe in several small studies in people with epilepsy (summarised in McDonald et al. (2018) and Borges (2022)). Our study in children included two teenagers with Dravet syndrome, who when adding triheptanoin to their diet both did not experience a consistent reduction in seizures (Calvert et al., 2018). A recent study of triheptanoin in 36 people with drug‐resistant GLUT1 deficiency found no seizure reductions in the total group, although people with only absence seizures had a 62% (median) reduction in seizures (Striano et al., 2022). Large phase III studies are needed to assess the anticonvulsant efficacy of triheptanoin in people with specific types of epilepsy.

### 5.6 Potential adverse effects of dietary metabolic therapies

For ketogenic diets, the main side effects that are experienced by people with epilepsy are constipation, emesis and abdominal pain (Kossoff et al., 2018; Neal et al., 2009). Other potential adverse effects include hyperlipidaemia, increased risk of skeletal fractures and kidney stones, and decelerated growth (Kossoff et al., 2018). For add‐on medium‐chain triglycerides (C8 and C10) and add‐on triheptanoin, the most common side effects are gastrointestinal and include diarrhoea, constipation, stomach cramps/abdominal pain, nausea and emesis (Borges et al., 2019, 2020; Calvert et al., 2018; Neal et al., 2009; Schoeler et al., 2021; Striano et al., 2022). These therapies are contraindicated in people with specific disorders that affect fat metabolism (Kossoff et al., 2018). Food intolerances and allergies, for example, to coconut, also need to be considered.

## 6 LIMITATIONS AND RECOMMENDATIONS FOR FUTURE RESEARCH

Evidence from human and/or animal studies suggest that metabolic changes occur in *SCN1A*‐ and *KCNA1*‐related epilepsies, which may create energy deficits and contribute to seizures. Below we highlight the methods and future experiments needed to fill major remaining knowledge gaps.

### 6.1 Brain harvest methods and metabolite levels

Using an optimal brain harvest method is critical to preserve in vivo concentrations of metabolites that are quickly metabolised and to produce results that accurately reflect that in living brain (Dienel, 2019b, 2020, 2021). Anaesthesia, which slows glucose metabolism, should be avoided if possible or considered in the interpretation (Flatt et al., 2021). ‘Snap‐freezing’ brain tissues via submersion into liquid nitrogen are common. However, from the time of death of the rodent or dissection during brain surgery until the tissue is frozen, enzymes are still active and degrade glucose, glycolytic and TCA cycle metabolites, while lactate accumulates. A post‐mortem interval of even 3–5 s can alter levels of certain metabolites in brain (Lowry et al., 1964; Ogushi et al., 1990). More suitable older brain harvest methods produce shorter post‐mortem intervals of 6–30 s in rodents but are technically challenging (Dienel, 2021). In our view and others, modern head‐focussed microwave fixation systems are the current ‘gold standard’, if they are available (Dienel, 2021; Juras et al., 2023). Head‐focused in situ microwave fixation results in near‐instantaneous death and complete brain fixation in <0.9 s in mice and ~1.4–1.7 s in rats. The rapid heating of brain tissue leads to near‐immediate inactivation of brain enzymes, and thereafter brains can carefully be removed and dissected. Also, microwave‐fixed tissue is unsuitable for assessing enzyme activities, ECAR and mitochondrial respiration, which are often measured alongside metabolites; however, assessment of gene expression and protein abundance is possible. Unfortunately, microwave fixation systems cannot be used if EEG electrodes are implanted; thus, simultaneous EEG recording and metabolite assessment are not possible.

A second related issue is that metabolite levels are often reported as relative amounts or peak areas, not actual concentrations. If standard curves and/or linearity of metabolite quantification assays have not been established, then questions arise regarding the truthfulness of the fold changes reported. In our experience and as raised by Dienel (2020), metabolite concentrations are important, as far departures from expected values indicate that the quality of the data is low. For instance, very high lactate concentrations in CNS tissue indicate that post‐mortem ischemia has occurred (Dienel, 2020), as we have found when studying metabolite levels in spinal cords, which are not properly fixed by head‐focussed microwaving (Tefera & Borges, 2019). Both relative and actual metabolite concentrations should be reported.

### 6.2 Functional techniques

Total metabolite levels, total protein abundances and mRNA expression of enzymes and other metabolic genes are of interest but do not provide information about function. To assess the function of metabolic pathways, metabolism of ^13^C‐labelled substrates can be tracked. If pathway rates are to be determined, the labelled substrates need to be administered continuously by intravenous infusion, until steady state is achieved (e.g. Marin‐Valencia et al. (2013)). A more straightforward approach is an intraperitoneal injection of the labelled metabolite and killing of rodents by microwave fixation after a fixed assay interval. In our work with ^13^C‐glucose (i.p.), we have used a 15‐min interval as this results in a relatively high label incorporation into glycolytic and TCA cycle intermediates as well as derived amino acids (~10–20%). Fifteen minutes also gives sufficient time to see incorporation into TCA cycle intermediates within a second turn of the cycle (e.g. McDonald et al. (2017, 2020)). Also, simultaneous tracking of ^13^C_1_‐glucose versus ^13^C_2_‐acetate metabolism can be used to distinguish and inform on global glucose metabolism versus astrocytic TCA cycle metabolism, as acetate is preferentially metabolised by astrocytes (e.g. Hassel et al. (1995), Melø et al. (2005)). Please note that when ^13^C_1_‐glucose versus ^13^C_2_‐acetate metabolism were administered i.v., the peak ^13^C‐incorporations into glutamate and glutamine via PDH metabolism of both ^13^C‐labelled substrates were achieved at around 15 min (Hassel et al., 1995). Alternatively, the metabolism of labelled substrates can be assessed ex vivo, in acutely isolated brain slices (McNair et al., 2017; Rae & Balcar, 2014; Westi et al., 2023). For this method, microwave fixation is not required and pharmacological inhibitors that do not cross the blood–brain barrier or are toxic to animals can be applied. However, a disadvantage of the brain slice approach is that some in vivo metabolite levels, such as glycogen, are lost post‐mortem and electrical activity and neuronal or glial signalling differ from that seen in vivo. Other modern approaches developed to study brain metabolism in vivo include genetically encoded metabolite sensors, for example, such as those developed for glucose and lactate, but are similarly not without caveats (Dienel & Rothman, 2024; Koveal, 2024). Finally, in vivo magnetic resonance spectroscopy studies with labelled substrates are very powerful, but they require high‐end equipment such as cyclotrons (e.g. Lai et al. (2018), Flatt et al. (2021), Rothman et al. (2021)).

### 6.3 Other limitations

It is important to note that brain metabolism is altered during and shortly after seizures. Thus, in animals with very high seizure frequencies, it can be difficult to distinguish between inter‐ and post‐ictal states. We also recognise that when metabolic treatments alter seizure frequency and/or severity, it is not possible to distinguish between direct metabolic changes and indirect effects of the treatment having altered the seizure frequency and/or severity, which also can affect metabolism. For instance, this is the case in our studies with triheptanoin and F16BP in the chronic mouse pilocarpine model of epilepsy (Hadera et al., 2014; McDonald et al., 2020, 2021).

## 7 SUMMARY AND FUTURE DIRECTIONS

Despite increasing awareness that brain metabolic dysfunction may play an important role in epilepsy (Kudin et al., 2009; Patel, 2018; Reid et al., 2014; Rho & Boison, 2022), only a small number of studies have investigated the metabolic aspects of genetic ion channel epilepsies. There is evidence of altered brain glucose metabolism in people with Dravet syndrome based on studies with FDG‐PET, but there are no human studies on brain metabolism in *KCNA1* epilepsy. The studies reviewed here in mouse and zebrafish models of these disorders indicate that metabolic changes occur, which may contribute to seizures. However, as pointed out throughout the article and particularly in Section 6, the interpretation of the studies was often difficult for various reasons. For example, energy metabolism is largely influenced by brain activity, which often was not or could not be assessed at the same time when mitochondrial respiration was assessed. On the other hand, the ketogenic diet is one of the most effective treatments for Dravet syndrome and has clear protective effects in animal models that lack *Scn1a/scn1Lab* and *Kcna1*. Overall, it may be surprising to some people within the epilepsy field that a metabolic therapy has efficacy in Dravet syndrome, considering that the primary cause of this epilepsy is genetic loss of function of SCN1A. In our opinion, it highlights that impairments in energy metabolism, which are consequences of genetic alterations, can play a major role and contribute to hyperexcitability in the brain. Thus, improving metabolism is a viable strategy to prevent seizures in epilepsies, even if metabolic alterations are not the primary cause. In addition, metabolic treatments often are preferred by patients and caregivers in lieu of medication and provide an alternative to genetic therapies that are not yet fully developed. Alternative dietary therapies such as add‐on medium‐chain triglycerides or triheptanoin may also be beneficial but require further investigation. To answer some of the unresolved questions regarding the metabolic changes seen in Dravet and *Kcna1*‐null mice, studies that measure metabolite levels including glycogen in microwave‐fixed brain tissue and track the metabolism of labelled glucose and other substrates in brain are expected to be useful.

## AUTHOR CONTRIBUTIONS

E.S.N. and K.B. conceived the idea for the review. E.S.N. produced the first draft of the text, figures and tables. E.S.N, W.X. and K.B. searched the literature, interpreted studies, and refined and finalised the manuscript. All authors read and approved the final version of the manuscript.

## FUNDING INFORMATION

We thank the National Health and Medical Research Council (grant number APP1186025) for the funding awarded to K.B., which supported E.S.N. and W.X., E.S.N. and W.X. are supported by PhD scholarships funded by the Commonwealth Government of Australia and The University of Queensland.

## CONFLICT OF INTEREST STATEMENT

K.B. has received consulting fees from Ultragenyx Pharmaceuticals Inc. and Nestlé Purina PetCare in the past. E.N. and W.X. declare no conflicts of interest.

### PEER REVIEW

The peer review history for this article is available at https://www.webofscience.com/api/gateway/wos/peer‐review/10.1111/jnc.15938.

## Data Availability

N/A
